# COVID-19 vaccine refusal is driven by deliberate ignorance and cognitive distortions

**DOI:** 10.1038/s41541-024-00951-8

**Published:** 2024-09-14

**Authors:** Kamil Fuławka, Ralph Hertwig, Thorsten Pachur

**Affiliations:** 1 Center for Adaptive Rationality, Max Planck Institute for Human Development, Berlin, Germany; 2 School of Management, Technical University of Munich, Munich, Germany

**Keywords:** Epidemiology, Epidemiology

## Abstract

Vaccine hesitancy was a major challenge during the COVID-19 pandemic. A common but sometimes ineffective intervention to reduce vaccine hesitancy involves providing information on vaccine effectiveness, side effects, and related probabilities. Could biased processing of this information contribute to vaccine refusal? We examined the information inspection of 1200 U.S. participants with anti-vaccination, neutral, or pro-vaccination attitudes before they stated their willingness to accept eight different COVID-19 vaccines. All participants—particularly those who were anti-vaccination—frequently ignored some of the information. This deliberate ignorance, especially toward probabilities of extreme side effects, was a stronger predictor of vaccine refusal than typically investigated demographic variables. Computational modeling suggested that vaccine refusals among anti-vaccination participants were driven by ignoring even inspected information. In the neutral and pro-vaccination groups, vaccine refusal was driven by distorted processing of side effects and their probabilities. Our findings highlight the necessity for interventions tailored to individual information-processing tendencies.

## Introduction

In 2019, the World Health Organization listed vaccine hesitancy—the reluctance or refusal to get vaccinated despite the availability of vaccines—as one of the top 10 global health threats^1^. Vaccine hesitancy is a complex phenomenon determined by historical, political, and socio-cultural factors, as well as individual knowledge and risk perception^2^. Recent reviews of over 100 surveys in high-, middle-, and low-income countries indicate that concerns about the side effects (risks) and effectiveness (benefits) of COVID-19 vaccines are among the main reasons for vaccine hesitancy^3,4^. Accordingly, many interventions to reduce vaccine hesitancy aim at providing factual information on *vaccine evidence*—that is, possible harms, potential benefits, and their probabilities—in a comprehensible fashion (e.g., using fact boxes)^5,6^. However, there is evidence that such transparent communication of the evidence does not impact people’s vaccination intentions^7^. Moreover, qualitative investigations show that the decision to refuse vaccination can be driven by factors unrelated to vaccine evidence, such as experiences of racism and mistreatment by medical professionals^8^, distrust of the pharmaceutical industry, or alternative understandings of medicine^9^. This begs the question of how (if at all) people use information about vaccine evidence. Do they ignore it? If they process it, are there distortions in the cognitive processing? Could the information be processed differently by people with different vaccination attitudes? And how does the effect of possible cognitive distortions on vaccine refusal compare to the effect of other relevant factors, such as demographic variables?

In this article, we leverage theoretical and analytical ideas as well as methodological tools from cognitive and behavioral science that have been developed to study individual decision processes to investigate how individuals with different attitudes toward COVID-19 vaccines process information on vaccine evidence. Our approach, where people make accept–refuse vaccination decisions for various existing COVID-19 vaccines (similar to refs. ^10,11^), allows us to characterize and measure how people process commonly provided information about vaccine evidence; it also allows us to capture and compare the influence of extraneous factors (which are unrelated to vaccine-specific information) on people’s vaccination decisions. Previous studies based on surveys and descriptive analyses—showing that people are more willing to accept a vaccine when it is more effective and has fewer and less frequent side effects—have not been able to cast light on these details of the decision process^10–19^.

**Fig. 1:**
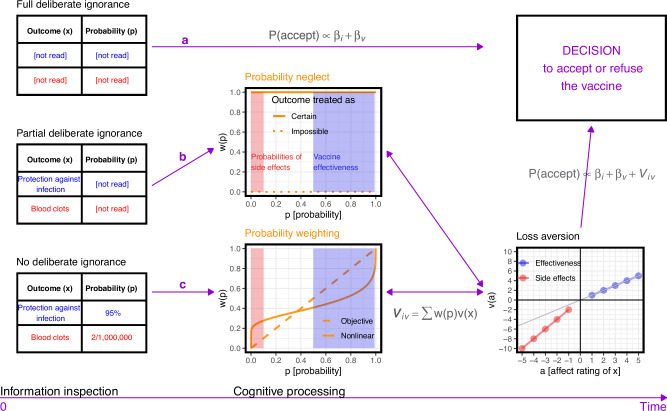
Information processing underlying vaccination decisions. Different ways of processing information about the vaccine evidence, yielding an individual *i*'s probability of accepting vaccine *v*, denoted by *P*(accept). Path **a** represents full deliberate ignorance. Vaccine evidence is not inspected at all, and the decision is based on other factors related to the individual *i* and the vaccine *v*, denoted by *β*_*i*_ and *β*_*v*_, respectively. Path **b** represents probability neglect, a type of partial deliberate ignorance in which only the possible outcomes of a vaccine but not their probabilities are acquired. In such cases, people usually perceive the outcome as certain to happen. However, in principle, it is also possible to ignore probability value and perceive the corresponding outcome as impossible to occur. Path **c** represents no deliberate ignorance. All information is inspected, but the probability information may be cognitively distorted via nonlinear probability weighting; the curvature of the probability weighting function measures the extent of such distortion. In paths (**b**) to (**c**), the neglected and weighted probabilities *w*(*p*) are integrated with the subjective values of the corresponding outcomes, which in the model are numerically represented by affect ratings *a*, transformed with value function *v*. The difference between the slopes of the value function over the side effects and the benefits of a vaccine constitutes a measure of loss aversion—a second cognitive distortion considered in our investigation.

We used process-tracing methodology and computational modeling to examine the extent to which people may engage in *deliberate ignorance*^20^ and how they may distort information on vaccine evidence during information processing. Figure 1 outlines our conceptual framework. In our study, we operationalize deliberate ignorance of vaccine evidence as choosing not to inspect a piece of information on a vaccine’s side effects, benefits, and their probabilities in the pre-decision phase. We distinguish three levels of deliberate ignorance: full, partial, and none. With *full deliberate ignorance*, people abstain from inspecting any information on vaccine evidence (Fig. 1a); their decisions may then be based on other factors instead, such as trust in the government or the belief that COVID-19 is no worse than a common cold (see refs. ^3,21,22^ for other factors). With *partial deliberate ignorance*, people ignore some—but not all—of the vaccine evidence information. Here, we focus on a specific manifestation of partial deliberate ignorance, *probability neglect* in which a vaccination outcome (e.g., side effect) is inspected, but its probability is not (Fig. 1b; see “Methods: Preregistration”). Probability neglect has been observed for dreadful risky outcomes^23,24^, including the side effects of medications^25–29^. These studies indicate that the neglected probability is treated as if the corresponding outcome was certain to occur, which, in case of outcomes such as vaccination side effects, would result in an increased rate of vaccine refusal (see Fig. 1b). Finally, with *no deliberate ignorance*, people inspect all information on vaccine evidence and consider it in their decision (Fig. 1c); even then, however, the cognitive processing of this information may be distorted (e.g., such that is it not fully considered in the decision) and thus deviate from what is considered a rational way to process information.

Vaccination decisions can be conceptualized as instances of risky choice^30,31^. Based on research into risky choice, our conceptual framework considers two types of cognitive distortions: (nonlinear) probability weighting and loss aversion. *Probability weighting* refers to the observation that people make risky decisions as if they processed probabilities nonlinearly, with low and high probabilities being over- and underweighted, respectively^32^. In vaccination decisions, this would mean overweighting the typically low probabilities of side effects and underweighting the typically high probabilities of benefits of a vaccine (Fig. 1, reduced probability sensitivity—stronger curvature of the probability weighting function indicates lower sensitivity). *Loss aversion* refers to the observation that people make risky decisions as if the psychological impact of losses is greater than that of gains^32^. In the context of vaccination decisions, loss aversion would mean that the psychological impact of possible side effects outweighs that of the potential benefits^33–35^. To capture loss aversion, comparable quantitative measures of the representations of the side effects and benefits are necessary. Medical outcomes often trigger pronounced self-reported^27,36–38^ and physiological affective reactions^27^; we therefore used positive and negative self-reported affect ratings of vaccines’ benefits and risks, respectively, to quantify people’s valuations of the outcomes. Loss aversion can then be measured by comparing the slopes of the value function over the side effects and the benefits of a vaccine, with a steeper slope over the side effects indicating loss aversion (Fig. 1).

**Fig. 2:**
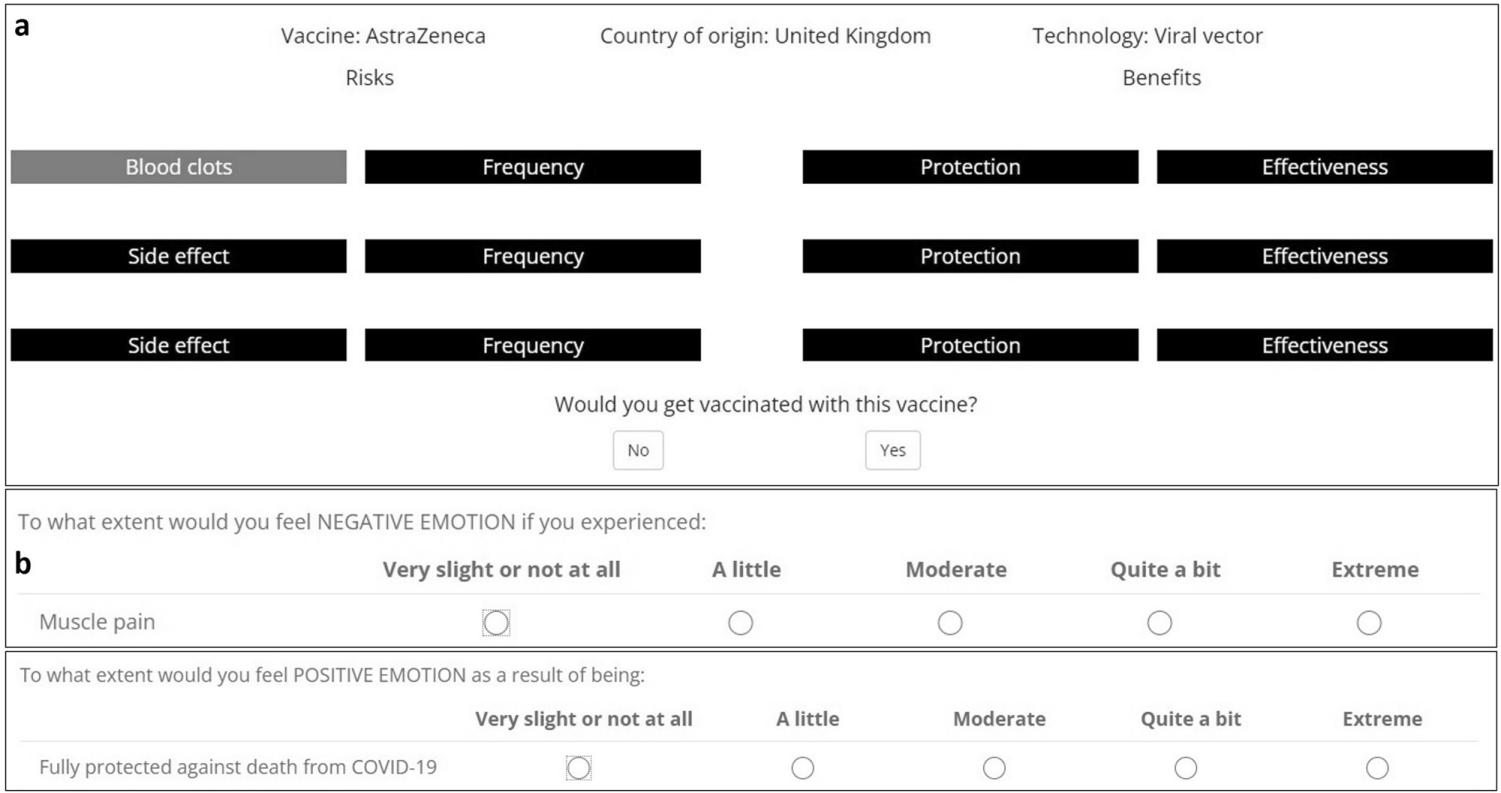
Experimental procedure. **a** Example screen from the decision task. Only one information box could be inspected (uncovered) at a time by hovering the mouse cursor over it. **b** Example items from the affect rating task. Participants rated their negative affect for the 15 side effects in a Likert matrix table: All side effects were presented at once, making it easier for participants to provide internally consistent ratings. Ratings of positive affect for the three benefits of vaccination were collected on a separate screen. Note that the labels for the different levels of the rating scales were identical for positive and negative affect. In the analyses, the positive and negative affect ratings were coded as 1 to 5 and −5 to −1, respectively.

To examine people’s information processing of vaccine evidence underlying COVID-19 vaccination decisions, we conducted an online study with U.S. citizens (*N* = 1200) who self-reported as having anti- (*n* = 365), neutral (*n* = 373), or pro- (*n* = 462) COVID-19 vaccine attitudes (vaccination attitude was measured using a single question with three response options; see “Methods: Study sample”). We recruited similarly large samples of anti-, neutral, and pro-vaccination participants to have comparable power when testing for potential processing differences between the three attitude groups (see “Methods: Preregistration”). Participants made a series of decisions about their willingness to get vaccinated with each of eight internationally licensed COVID-19 vaccines, one after the other. For each vaccine, participants could choose to inspect information on vaccine evidence, including side effects (e.g., blood clots, severe headache, tiredness) and benefits (protection against COVID-19 infection, severe illness, and death and the corresponding probabilities) (see Table 1). We recorded participants’ information inspection behavior—choosing to view a piece of information—with the process-tracing methodology Mouselab^39^ (see “Methods: Mouselab task”). In Mouselab, the attributes of objects—here, pieces of information on vaccine evidence—are hidden behind labeled boxes, and each attribute can be inspected, one at a time, by hovering the mouse cursor over the respective box (Fig. 2a). In each trial, the brand, vaccine technology, and country of development were clearly displayed at the top of the screen. Participants could decide based on this information without inspecting any information about the vaccine evidence. Participants could explore the available information as long and often as they wished before deciding whether to accept or refuse the vaccine. Finally, we obtained quantitative measures of each participant’s subjective valuations of the outcomes by asking them to provide affect ratings for each side effect and benefit (Fig. 2b; see also “Methods: Affect rating task”).

## Results

In all statistical analyses, we used Bayesian hierarchical models with participant-level intercepts—accounting for each participant making eight decisions—and the analyses were always conducted on the level of a single decision (see “Methods: Statistical modeling”). Our statistical inferences were based on the posterior distributions of the regression weight of interest (e.g., the difference between attitude groups in vaccine acceptance rates). For each dependent variable, we present the model-based predicted value of the variable (e.g., the posterior predictive distribution of vaccine acceptance probability) and the raw data (e.g., the observed vaccine acceptance proportions). The predicted values shown in the figures are medians of the posterior predictive distributions with 95% highest density intervals (HDI).

### Vaccination decisions

A total of 61.9% (226/365) of participants in the anti-vaccination group, 11.7% (44/373) of participants in the neutral group, and 0.4% (2/462) of of participants in the pro-vaccination group refused all eight vaccines. On average, participants in the anti-vaccination, neutral, and pro-vaccination groups accepted one, three, and five of the eight vaccines, respectively. Interestingly, the non-zero acceptance rate in the anti-vaccination group was mainly driven by almost 30% of these participants indicating a willingness to accept the Indian vaccine Bharat Biotech.

**Fig. 3:**
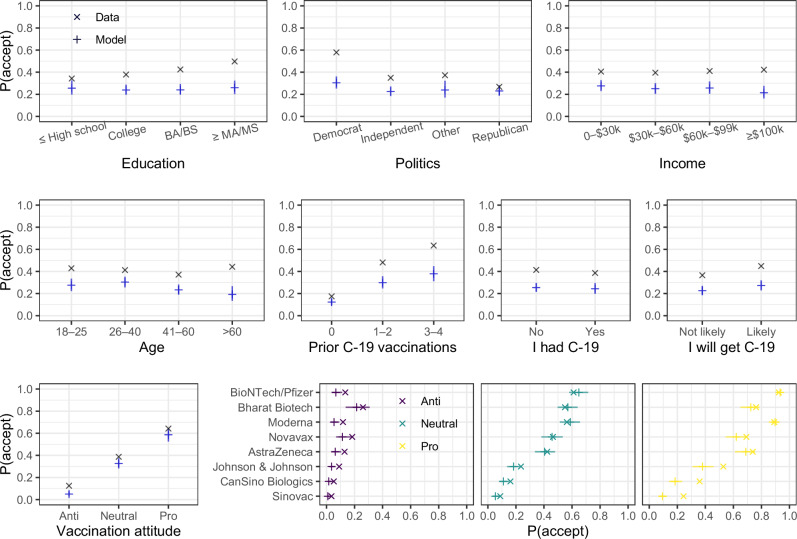
COVID-19 vaccine acceptance rates by demographic variables and separately for each vaccine. The × symbols show empirical data; the + symbols show the posterior predictive acceptance probabilities from a Bayesian hierarchical logistic regression containing all presented variables. The horizontal line represents the median of the posterior predictive distribution, and the vertical line represents the 95% highest density interval.

We first tested how demographic and individual variables were related to the decision to accept or refuse a vaccine (Fig. 3). The strongest predictors of vaccine acceptance were vaccination attitude, the number of vaccinations against COVID-19 a participant had received by the time of the study, and vaccine brand. The raw data showed that political orientation and education level were related to vaccination decisions—consistent with results in previous studies (see ref. ^3^). These relationships, however, vanished when tested in the full statistical model, because political orientation and education (and age) had strong relationships with vaccination attitudes and the history of vaccinations against COVID-19 (see Supplementary Information). The strong link between participants’ vaccination decisions measured in our study and their vaccination attitudes and actual vaccination history suggests that they took the decision task seriously.

We conducted two main sets of analyses to investigate the information processing underlying participants’ vaccination decisions. First, we used statistical models to investigate the relationship between deliberate ignorance of vaccine evidence (measured with Mouselab) and vaccination decisions. Second, we used computational modeling to investigate the cognitive distortions in the processing of the inspected vaccine evidence—that is, probability weighting and loss aversion—and its impact on vaccination decisions.

### Deliberate ignorance and vaccination decisions

Our first main set of analyses focused on how participants’ vaccination decisions were related to whether they inspected all, some, or no information on vaccine evidence. We begin by analyzing full and partial deliberate ignorance and then examine probability neglect (a specific case of partial deliberate ignorance) in greater detail (see “Methods: Preregistration”).

In the next two sections, we report results from statistical models that included demographics and individual variables (Fig. 3) as covariates. In the Supplementary Information, we report robustness checks testing whether the inclusion of these covariates or the selection of specific subsets of covariates affects our conclusions. We note in the main text which results were not robust.

#### Did participants engage in deliberate ignorance, and how was it related to vaccination decisions?

Drawing on the data recorded with Mouselab, we analyzed participants’ information inspection behavior by assigning each trial to one of three levels of deliberate ignorance (see “Methods: Preprocessing of information inspection data”): (1) full deliberate ignorance—the vaccine evidence was not inspected at all, (2) partial deliberate ignorance—some but not all of vaccine evidence information was inspected, and (3) no deliberate ignorance—each piece of information on vaccine evidence was inspected at least once.

**Fig. 4:**
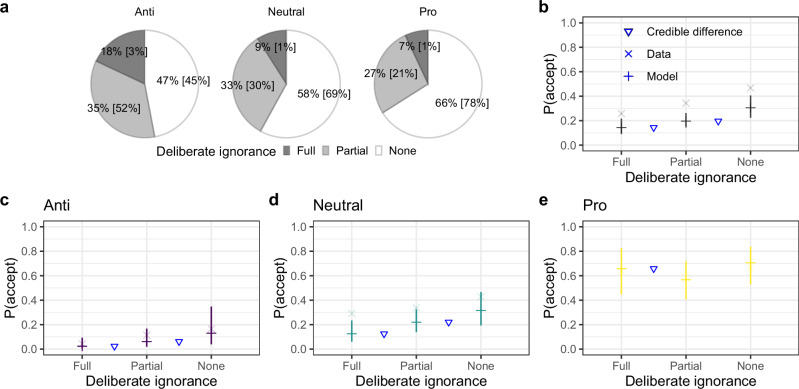
Deliberate ignorance and vaccination decisions. **a** Proportions of vaccination decisions preceded by different levels of deliberate ignorance, separately for the three attitude groups. Pie charts show the raw data; values in brackets are medians of the posterior predictive distributions from Bayesian hierarchical ordered-logit regression. **b**–**e** Relationships between the levels of deliberate ignorance and vaccine acceptance in the entire data set (panel **b**) and separately for the anti-vaccination, neutral, and pro-vaccination participants. The × symbols show empirical data. The + symbols indicate the posterior predictive acceptance probabilities from Bayesian hierarchical logistic regressions—the horizontal line represents the median of the posterior predictive distribution, and the vertical line represents the 95% highest density interval. A triangle indicates when the 95% highest density interval of the posterior difference between the neighboring estimates excluded zero.

As shown in Fig. 4a, anti-vaccination, neutral, and pro-vaccination participants exhibited full deliberate ignorance in 18%, 9%, and 7% of decisions, respectively. A comparison of the proportions of decisions with different levels of deliberate ignorance across the three participant groups showed that anti-vaccination participants ignored information to a larger extent than neutral (Δ = −1.03, 95% HDI: [−1.56, −0.51]) or pro-vaccination participants (Δ = −1.45, 95% HDI: [−2.19, −0.77]); the proportions did not differ between the neutral and pro-vaccination groups (Δ = −0.43, 95% HDI: [−0.16, 1]).

The level of deliberate ignorance was strongly related to vaccination decisions: The probability of vaccine refusal was highest when participants exhibited full deliberate ignorance and lowest when participants exhibited no deliberate ignorance (Fig. 4b). This aggregate pattern also held within the anti-vaccination and neutral groups (Fig. 4c–d). In the anti-vaccination group, full deliberate ignorance was almost always followed by vaccine refusal; in the pro-vaccination group, by contrast, full deliberate ignorance was associated with a higher probability of vaccine acceptance than partial deliberate ignorance. This suggests that in the pro-vaccination group, full deliberate ignorance was often driven by general and external factors such as trust in science and institutions that—from the point of view of these participants—made the inspection of information about vaccine evidence superfluous.

#### How was probability neglect related to vaccine refusal?

We defined probability neglect as cases in which there was at least one instance where a participant inspected an outcome but not its probability (see “Methods: Preprocessing of information inspection data”). Thus, probability neglect was indexed with a binary variable on the level of each trial: Probability neglect (1) occurred or (2) did not occur before the decision was made. We created separate indices of probability neglect for side effects and for benefits; we also measured probability neglect for side effects depending on the severity of the side effect (i.e., extreme, severe, or mild; see “Methods: Vaccine evidence data”).

**Fig. 5:**
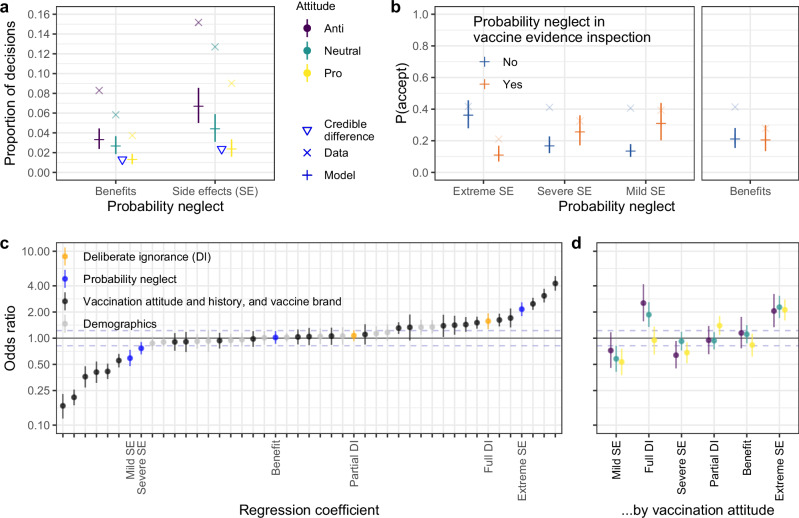
Probability neglect and vaccination decisions. **a** Occurrence of probability neglect in information inspection, analyzed separately for side effects and benefits. A triangle indicates when the 95% highest density interval of the posterior difference between the neighboring estimates excluded zero. **b** Vaccine acceptance rates as a function of the type of probability neglect. In (**a**) and (**b**), the × symbols show empirical data. The + symbols show posterior predictive probabilities from Bayesian hierarchical logistic regressions—the horizontal line represents the median of the posterior predictive distribution, and the vertical line represents the 95% highest density interval. **c** Odds ratio values from a Bayesian hierarchical logistic regression. All factors were coded with sum-to-zero contrasts, such that the coefficients indicate the deviation from the average vaccine acceptance rate. The coefficients for vaccination attitude and history, and vaccine brand represent the effects of vaccination attitude, vaccine brand, and the interaction of the two; vaccination history refers to the effect of the number of actual vaccinations against COVID-19. The area outside the dashed lines is a space of effect size values considered practically relevant by convention (i.e., $| \log (OR)| \ge 0.2$). **d** Odds ratio values from Bayesian hierarchical logistic regressions, estimated separately for each attitude group. The odds ratios for the predictors deliberate ignorance and probability neglect in panels (**c**) and (**d**) are the ratios for vaccine refusal.

Participants in the anti-vaccination, neutral, and pro-vaccination groups exhibited probability neglect for side effects in 15%, 13%, and 9% of vaccination decisions, respectively, and for benefits in 8%, 6%, and 4% of decisions, respectively. Furthermore, 47%, 40%, and 31% of participants in the anti-vaccination, neutral, and pro-vaccination groups, respectively, exhibited probability neglect for side effects in at least one vaccination decision; for benefits, the figures were 33%, 29%, and 19%, respectively. Nominally, both probability neglect for side effects and probability neglect for benefits were more frequent in the neutral and anti-vaccination groups than in the pro-vaccination group (Fig. 5a), but these differences were not robust (see Supplementary Information).

To examine how probability neglect for side effects and benefits was related to vaccination acceptance or refusal, we tested them as predictors of vaccination decisions. Probability neglect for side effects was linked with an increased probability of vaccine refusal (OR_refuse_ = 1.22, 95% HDI: [1.09, 1.35]), as was probability neglect for benefits (OR_refuse_ = 1.22, 95% HDI: [1.05, 1.42]), but the latter result was not robust (see Supplementary Information).

The effect of probability neglect on vaccination decisions may also depend on the severity of the side effect^40^. We therefore distinguished whether the probability neglect occurred for an extreme, severe, or mild side effect or for a benefit. The analysis revealed two robust effects (Fig. 5b). First, vaccine refusal was much more likely in trials where the probability of an extreme side effect was neglected (Fig. 5b–d). For example, participants who learned that AstraZeneca could lead to blood clots but did not learn that the probability of this side effect is extremely low were much more likely to refuse the vaccine than those who inspected both pieces of information. Second, vaccine refusal was much less likely in trials where the probability of a mild side effect was neglected. For instance, participants who learned that Sinovac could lead to tiredness but did not learn that this side effect occurs frequently were much more likely to accept the vaccine than those who inspected both pieces of information. This result makes intuitive sense but is at odds with the existing literature, which has focused only on how probability neglect leads to the avoidance of potentially dangerous events^23,24^.

The effects of probability neglect for extreme and mild side effects, and the effect of full deliberate ignorance, were substantially larger than the effects of the demographic variables and were among the strongest effects in the regression model (Fig. 5c). The three effects also held across vaccination attitude groups (Fig. 5d) and more than 30 different model specifications (see Supplementary Information). Thus, how participants inspected and ignored information about vaccine evidence seemed to be a key predictor of their decision to get vaccinated with a given vaccine or not.

### Cognitive distortions of vaccine evidence

In the next set of analyses, we used computational modeling to investigate the cognitive distortion of information on the vaccine evidence that participants inspected. Recall that in order to have a quantitative measure of participants’ subjective valuation of a vaccine’s possible outcomes, we asked each participant to rate (see Fig. 2b) the negative (positive) emotion they would feel due to each side effect (benefit). Here, we start by comparing the average affect ratings across the three attitude groups. We then used the individual affect ratings as numerical inputs in computational modeling to investigate how the inspected information on vaccine evidence, including probabilities, was processed.

#### Did affect ratings of vaccine outcomes vary by vaccination attitude?

**Fig. 6:**
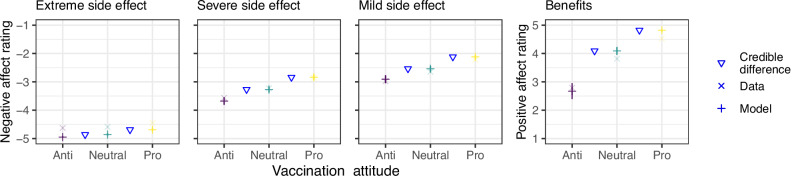
Affect ratings of vaccination outcomes. The × symbols show empirical data. The + symbols show expected mean affect ratings from Bayesian hierarchical ordered-logit regressions, estimated separately for each outcome group—the horizontal line represents the median of the posterior predictive distribution, and the vertical line represents the 95% highest density interval. A triangle indicates when the 95% highest density interval of the posterior difference between the neighboring estimates excluded zero.

The average affect ratings of the anti-vaccination, neutral, and pro-vaccination groups are presented in Fig. 6 (see “Methods: Statistical modeling”). The anti-vaccination group gave the most negative affect ratings for side effects and the least positive ratings for benefits. The pro-vaccination group gave the least negative affect ratings for side effects and the most positive ratings for benefits. The affect ratings of the neutral group fell in between. Differences in average affect ratings between groups were much more pronounced for benefits (i.e., positive affect ratings) than side effects (i.e., negative affect ratings). These results show that vaccination attitudes are themselves associated with different emotional reactions to the possible outcomes of a vaccine that are independent of the vaccine brand or other external factors. All groups rated severe side effects as less negative than extreme side effects and more negative than mild side effects; this shows that the affect ratings track the objective magnitudes of the side effects and suggests that they constitute a reasonable measure of the subjective values of the vaccination outcomes for use in the computational modeling.

#### Nonlinear probability weighting and loss aversion in vaccine decisions

We now turn to how participants processed the information about vaccine evidence that they inspected before making a vaccination decision. To this end, we developed a computational model (see “Methods: Computational modeling”) that can capture all paths to reach a decision presented in Fig. 1. In the model, the probability of individual *i* accepting a vaccine *v*, denoted by *P*(accept), is a function of three additive components:

1 $$P(\,\text{accept}\,)\,\propto {\beta }_{i}+{\beta }_{v}+\varphi {V}_{i,v}.$$

The *β*_*i*_ parameter is an individual-level decision bias and captures a participant’s general propensity to accept or refuse a vaccine, irrespective of the vaccine’s properties. The *β*_*v*_ parameter captures effects that are specific to a given vaccine, such as country of origin or the underlying technology, and do not pertain to the vaccine evidence; these effects are assumed to be the same for all participants. Finally, the term *φV*_*i*,*v*_ represents a model-based estimate of participant *i*’s subjective valuation of vaccine *v*. As shown in Fig. 1b–c, this valuation is based on vaccine evidence information—the vaccine’s outcomes, numerically represented by the individual affect ratings, and their probabilities—and is derived using prospect theory^32^ (see “Methods: Formal model specification”).

**Fig. 7:**
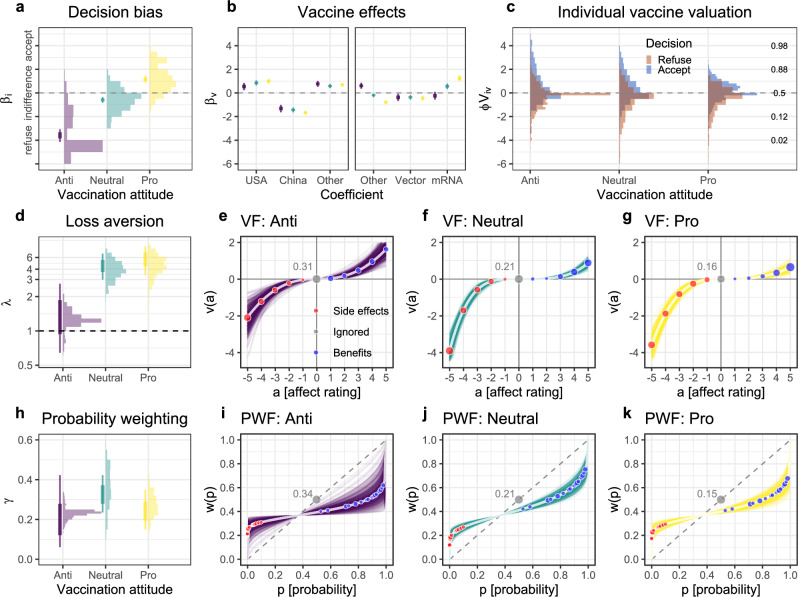
Information weighting and distortion. **a** Group-level (vertical lines) and individual-level (histograms) decision biases. **b** Group-level estimates of vaccine-specific effects on vaccination decisions. The coefficients show the predicted difference in the probability of refusing or accepting a vaccine from the average probability in a given group. **c** Distributions of subjective valuations *φV*_*i*,*v*_, separately for accept and refuse decisions. The *y*-axis in panels (**a**–**c**) is on the scale of the linear predictor, which is then transformed via the inverse-logit function to obtain the predicted probability of vaccine acceptance shown on the right of panel (**c**). **d** Group-level (vertical lines) and individual-level (histograms) estimates of the loss aversion parameter *λ*. *λ* > 1 values indicate loss aversion; *λ* = 1 indicates loss neutrality. **e**–**g** Value function (VF) for the anti-vaccination, neutral, and pro-vaccination participants based on the group-level posterior distributions of the parameter estimates. **h** Group-level (vertical lines) and individual-level (histograms) estimates of the probability weighting parameter *γ*. Higher values of *γ* indicate more linear probability weighting. **i**–**k** Probability weighting function (PWF) for the anti-vaccination, neutral, and pro-vaccination participants based on the group-level posterior distributions of the parameter estimates. In panels (**a**, **d**, and **h**), the thin and thick vertical lines show 95% and 80% highest density intervals, respectively, of the posterior distribution of the group-level parameter estimates. The histograms show distributions of the posterior means of the individual-level parameters. The gray values and points in panels (**e**–**g**) and (**i–k**) give the proportions of ignored outcomes and probabilities, respectively, within each attitude group.

To what extent were the vaccination decisions driven by individual decision biases, vaccine-specific effects, and subjective distortion of vaccine evidence? The first row in Fig. 7 addresses this question. For the majority of the anti-vaccination group, there was a decision bias (Fig. 7a) to refuse the vaccine that was so strong that the effects of the vaccine’s properties (Fig. 7b) and valuations (Fig. 7c) rarely pushed the probability of acceptance above 50%. In the neutral group, the distribution of the decision bias was clustered below zero (Fig. 7a), indicating that these participants showed a weak a priori propensity to refuse a vaccine; nevertheless, their vaccination decisions were also driven by vaccine-specific effects (Fig. 7b) and by consideration of vaccine evidence information (Fig. 7c). Almost all participants in the pro-vaccination group showed a bias toward accepting the vaccine (Fig. 7a), but the size of this bias was not as large as the refusal bias in the anti-vaccination group. Overall, the distributions of subjective valuations *V*_*iv*_ of the vaccine evidence (Fig. 7c) were comparable to the distributions of the individual decision biases. This indicates that the subjective valuations of the vaccine’s effectiveness, side effects, and probabilities drove the vaccination decisions, particularly among the neutral and pro-vaccination participants.

The value function captures the subjective valuation of the vaccines’ side effects and benefits (Fig. 7d–g). These results show that nearly all participants exhibited substantial loss aversion. This is indicated by the individual-level values of the *λ* parameter above 1 (Fig. 7d) and the asymmetric shapes of the curves over the ratings of side effects and benefits (Fig. 7e–g). On average, the impact of a side effect rated as extremely negative was four times stronger than that of a benefit rated as extremely positive among the neutral and pro-vaccination participants. In the anti-vaccination group, the average degree of loss aversion was lower. Although this may seem counterintuitive, note that the anti-vaccination participants already expressed considerably stronger affective responses to the side effects than to the benefits (Fig. 6).

The probability weighting function (Fig. 7h–k) captures the subjective valuation of probabilities, and the function’s shape is governed by the probability weighting parameter *γ*_*i*_, with higher values indicating more linear probability weighting (and lower values indicating lower sensitivity to differences in probability). On average, individual probability weighting *γ*_*i*_ was highest in the neutral group and lowest in the anti-vaccination group. Additional analyses suggest that anti-vaccination participants ignored probabilities even after inspecting them (see Supplementary Information). The inverse S-shaped form of the estimated probability weighting functions indicates that overall, the impact of small probabilities of side effects on vaccination decisions was higher than warranted based on their objective value; the impact of high probabilities of protection was smaller than warranted based on their objective value.

Qualitatively, both the value and probability weighting functions show relatively similar patterns across the three groups: All participants exhibited some degree of loss aversion and nonlinear probability weighting. Thus, computational modeling suggests that the vaccination decisions of all groups were characterized by some sort of aversion to side effects: Negative affect associated with side effects had more impact on vaccination decisions than did the positive affect associated with benefits (protection), probabilities of side effects were overweighted, and probabilities of benefits were underweighted. Since neutral and pro-vaccination participants were the most sensitive to vaccine evidence, these results suggest that cognitive distortion in the form of side-effect aversion was among the main reasons for vaccine refusal decisions in these two groups.

## Discussion

According to principles of good evidence communication^41^, the overarching aim should be to inform rather than persuade. This means, for instance, not cherry-picking findings and results but rather presenting “potential benefits and possible harms in the same way so that they can be compared fairly” (see p. 363 in ref. ^41^. In light of these objectives of evidence communication, however, it is also crucial to understand the effects of evidence communication. For instance, how do people process evidence about the potential benefits and harms of vaccines that are provided to them? We used process-tracing methodology and computational modeling to investigate how participants with anti-vaccination, neutral, and pro-vaccination attitudes inspected and processed evidence about COVID-19 vaccines: Did they fully inspect all evidence or deliberately ignore some of it^20^? And if they deviated from rational information processing, in what way?

All three attitude groups deliberately ignored some or all vaccine evidence information. Exhaustive inspection of the evidence was associated with higher vaccine acceptance. By contrast, inspecting information about possible extreme side effects but not their probabilities—an instance of probability neglect—was strongly associated with the decision to refuse a vaccine. Participants in all three groups valued the risks and benefits of vaccines unequally, showing aversion to side effects—in the sense that they had a stronger psychological response to the possible side effects of vaccines than to their potential benefits (akin to loss aversion in choices between risky prospects). In addition, all three groups overweighted the low probabilities of side effects, albeit to a different extent (see also refs. ^33,35^).

We also observed important differences between the groups in information processing. A substantial proportion of participants in the anti-vaccination group did not inspect any evidence about the vaccines. This full deliberate ignorance of vaccine evidence was almost always associated with vaccine refusal. Furthermore, the computational modeling analysis suggested that the anti-vaccination group’s high refusal rate was driven by a strong decision bias against vaccination. This means that in this group, the decision to refuse vaccination was essentially insensitive to evidence about the COVID-19 vaccines, even if evidence was initially inspected. There could be various reasons for this pronounced bias against vaccination, including mistrust in government, science, doctors, and health authorities^42,43^. Indeed, in the absence of basic trust, evidence about vaccines may be deemed to lack credibility. Participants with a vaccination-neutral attitude—who probably represent the largest group in the population^44^—displayed the most “rational” information processing. They showed the most linear probability weighting and overall highest sensitivity to vaccine evidence. Furthermore, they were not more likely to deliberately ignore vaccine evidence information than those in the pro-vaccination group.

Several insights follow from our findings. They underscore the importance of tailoring interventions to increase vaccine uptake to specific target groups. Our findings suggest a simple way to predict a person’s processing of information about vaccine evidence—namely, based on their attitude to COVID-19 vaccination. This can be measured by asking people to categorize themselves as pro-vaccination, neutral, or anti-vaccination. A person’s general vaccination attitude could be assessed before medical interviews or implemented on information websites. Based on the self-declared attitude, the content and format of the presented information could be adjusted accordingly (e.g., whether it is necessary first to establish trust or whether the focus should be on making risk information more accessible). However, this idea requires further testing, including how exactly people understand the ‘neutral’ vaccination attitude and how exactly to tailor the interventions to neutral and anti-vaccine participants. In the following paragraphs, we offer concrete ideas on how such tailored interventions could look like.

The prevalent deliberate ignorance of vaccine evidence among the anti-vaccination constitutes a practical barrier to the approach of risk communication that is meant to inform but not persuade^41^. Importantly, however, this does not mean that health communicators and health authorities tasked with evidence communication should abandon the goal of informing and instead give in to the urge to persuade or oversimplify (see ref. ^41^). Instead, it means that risk evidence communicators need to be realistic about their expectations. It also means that they must consider other aspects of their efforts, such as the relationship between the communicator and the audience (e.g., it may be advisable to deploy trusted community-based vaccination champions who are willing and able to engage in dialogue and support communication activities^45^). In addition, it means explicitly addressing the major concerns of segments of the population that are skeptical of vaccination. This includes disclosing uncertainties and addressing what science does not know^41,46^; communicating about side effects and adverse events in understandable, nontechnical, and transparent language to maintain trust and counter misinformation^5,47^; and explicitly addressing vaccination-related myths and false information (see ref. ^45^).

Assuming that trust can be re-established among people with an anti-vaccination attitude, how will they process the evidence when they inspect it? Here, the results of our modeling approach are particularly relevant. In the anti-vaccination group, we observed that individuals, to the extent that they inspected the vaccine evidence, based their decisions solely on possible side effects and benefits (e.g., the protection against severe COVID-19) but disregarded the probabilities, even if they were inspected (for further details, see Supplementary Information). This tendency to close one’s eyes to probabilities is qualitatively different from the information processing in the group of neutral participants. Based on these observations, targeted interventions that address this disregard of probabilities (including the overweighting of the side effects’ small probabilities) appear desirable.

What shape might such an intervention take? One approach is to use interactive simulations to convey vaccine evidence. Such simulations imitate the sequential and experiential mechanisms by which people naturally encounter risk information. A recent study^48^ found that vaccination-hesitant adults were more likely to express an intention to get vaccinated when they learned about the likelihood of experiencing various COVID-related events with and without vaccination through interactive simulation relative to being presented with the same information in fact boxes. One possible reason for this effect may be the inescapable sampling involved in the interactive risk simulation. This may make it more difficult to ignore probability information (e.g., of side effects), leading to less deliberate ignorance and less distorted probability weighting. In addition, there is evidence that presenting probabilities in visual formats such as icon arrays, where icons represent people affected versus not affected, can reduce side-effect aversion^34^. Importantly, interactive simulations may also be a pertinent method to address the aversion to side effects in the group of people with neutral attitudes.

More generally, side-effect aversion occurred across all three groups. All groups valued possible risks higher than potential benefits (see Fig. 7d–g). To avoid these strong reactions to side effects, authorities might be tempted not to disclose them or to disclose them selectively for fear of jeopardizing public vaccine acceptance. While such a policy may initially decrease vaccine hesitancy, it comes at a huge cost: Limited transparency undermines trust in health authorities and fosters the spread of conspiracy beliefs^47^. How is it possible to provide full transparency without prompting disproportionate overweighting of side effects or partial ignorance? One option is interactive simulations (described above); another is targeting the strong negative emotions associated with side effects. A recent multi-country study found that cognitive reappraisal (i.e., changing how one thinks about a situation) is an effective strategy for reducing negative emotions in the context of COVID-19^49^. By extension, cognitive reappraisal may also be effective in reducing negative emotions about vaccines. For example, the ‘rethinking’ strategy included in ref. ^49^ study could involve putting vaccination side effects into a broader perspective by emphasizing that any side effects would be less severe than COVID-19 symptoms without vaccination, or that extreme adverse events such as blood clots are in fact much more likely after a COVID-19 infection than after a vaccination—and that even when they do occur, the chances for successful treatment are very high.

We note a couple of limitations of our study. First, participants were paid a flat rate upon completion of the survey (see “Methods: Study sample”). This incentive scheme may have led some participants to click through the study as quickly as possible and collect the payment. However, given that Prolific participants have been shown to be of high quality^50,51^, and given our observation of systematic patterns of information inspection and processing across the three groups, it seems unlikely that such minimal-effort behavior critically shaped our results. Second, we did not explore people’s reasons for deliberate ignorance. Even though we instructed our participants to assume that the presented vaccine evidence is, in principle, applicable to them personally (see “Methods: Mouselab task”), some individuals may have engaged in deliberate ignorance because they thought of themselves as already fully informed about the evidence. If this was the case, however, participants should be more likely to ignore evidence related to the U.S.-approved vaccines but inspect evidence about the other, less familiar vaccines. However, we observed nearly as much deliberate ignorance with regard to vaccines approved for the U.S. as for those not approved: on average, 46% and 41%, respectively. Based on these numbers, it seems safe to conclude that preexisting knowledge of vaccine evidence was probably not among the main reasons for not inspecting the vaccine evidence in our investigation.

Relatedly, our results do not establish a causal link between deliberate ignorance and vaccination decisions but rather reveal associations between them. A widely discussed explanation for the public divide in beliefs about, for instance, vaccination, climate change, or evolution is that people engage in ‘motivated reasoning’^52^. According to this argument, individuals skeptical about vaccination or climate change are inclined to reject ostensibly credible scientific information because it contradicts their prior beliefs. Rejecting scientific evidence to thus protect one’s beliefs (or even one’s identity) could take the form of not inspecting it altogether or inspecting only parts of it. It is possible that belief-protecting or identity-protecting processing underlies acts of deliberate ignorance (multiple causes have been suggested and discussed; see ref. ^53^). It is an exciting avenue for future research to investigate in more detail the reasons behind the information processing observed in the anti-vaccination group—such as motivated reasoning or suspicion of medical services caused by experiences of racial discrimination by public institutions. Finally, the strength of vaccine hesitancy is likely to vary across people. We have simplified its measurement so as to avoid complicating the complex modeling across groups. Future studies could use a more fine-grained measure of vaccine hesitancy, potentially providing further insights into the relationship between hesitancy and the processing of vaccine evidence.

Developing and manufacturing safe and effective COVID-19 vaccines within a year was a breathtaking success story of human ingenuity. Yet this story had a sobering aftermath: Worldwide, many people refused vaccination. It is now the task of behavioral scientists to understand the reasons for this phenomenon and to design better ways of communicating the available evidence on the risks and benefits of COVID-19 vaccines. Our findings that people often deliberately ignore vaccine evidence or process it in ways counter to rational standards suggest that effective evidence communication must take new and innovative paths. Societies can be fully prepared for future pandemics only when technological ingenuity is coupled with cognitive and behavioral insights.

## Methods

### Study sample

We used Prolific and its filtering options to collect complete data from 1200 U.S. adults and obtain a relatively balanced sample of participants with anti-vaccination, neutral, and pro-vaccination attitudes. We created three instances of the same study, each available to 400 participants. One was run with participants with anti-vaccination attitudes, another with participants with pro-vaccination attitudes, and a third with participants with neutral vaccine attitudes, as declared in the survey that Prolific provides to all platform users. At the end of the study, we asked participants about their vaccination attitudes to check for possible changes since the initial measurement by Prolific. Specifically, we asked “How would you characterize your general attitude toward vaccination against COVID-19? [Against, Neutral, Pro].” Most observed changes were in the pro-vaccination direction, and the final sample consisted of 365 anti-vaccination, 373 neutral, and 462 pro-vaccination participants. For all our analyses, we used the attitude that the participants reported at the end of the study.

The sample consisted of 720 women (60%), 463 men (39%), and 17 participants who chose “other” as their gender. The mean age was 38.23 years (SD = 13.76). We estimated that participants would need up to 20 minutes to complete the study; the actual median completion time was around 12 minutes. Participants were remunerated with 2.50 GBP. See Supplementary Fig. 1 for the distribution of all collected demographic variables within each attitude group.

The study was approved by the Internal Review Board (Ethics Committee) of the Max Planck Institute for Human Development. To participate in the study, each participant had to provide informed consent by accepting the terms and conditions outlined in the Study Information and Statement of Informed Consent for Adult Participants, presented at the beginning of the procedure. Data collection occurred between April 19 and April 25, 2022.

### Vaccine selection

We included eight existing COVID-19 vaccines in the decision task, asking participants to state their willingness to accept or refuse vaccines (see Fig. 2 and “Methods: Mouselab task”). There were several reasons why we included more vaccines than only the three available in the U.S. First, we included multiple vaccines to be able to obtain a more precise measurement of individual tendencies to refuse or accept a vaccine. Second, the different vaccines differed in terms of their worst side effect and their effectiveness statistics (see Table 1); including these vaccines allowed us to get more precise estimates of the tested effects of probability neglect, loss aversion, and probability weighting. Third, we considered it relevant to include vaccines that are based on different technologies and/or were developed in different countries, as we wanted to measure to what extent participants’ decisions were sensitive to factors other than vaccination risks and benefits. Finally, including vaccines not available in the US reduced the possibility that people already had extensive knowledge of the vaccines’ risks and benefits.

**Table 1:** Data presented in the Mouselab task

	Side Effects (Risks)					
	Side effect 1	Side effect 2	Side effect 3	*P* (Side effect 1)	*P* (Side effect 2)	*P* (Side effect 3)
AstraZeneca	Blood clots	Immune system attacks nerves	Immune system attacks blood	20 out of 1,000,000	10 out of 1,000,000	5 out of 1,000,000
BioNTech/Pfizer	Heart muscle inflammation	Heart membrane inflammation	Severe tiredness	16 out of 1,000,000	28 out of 1,000,000	46,000 out of 1,000,000
Moderna	Heart muscle inflammation	Heart membrane inflammation	Severe muscle pain	25 out of 1,000,000	32 out of 1,000,000	100,000 out of 1,000,000
Johnson & Johnson	Blood clots	Immune system attacks nerves	Severe muscle pain	38 out of 1,000,000	98 out of 1,000,000	14,000 out of 1,000,000
Novavax	Severe general discomfort	Severe tiredness	Severe muscle pain	63,000 out of 1,000,000	83,000 out of 1,000,000	49,000 out of 1,000,000
Sinovac	Facial paralysis	Tiredness	Muscle pain	50 out of 1,000,000	82,000 out of 1,000,000	40,000 out of 1,000,000
Bharat Biotech	Fever	Headache	Tiredness	6700 out of 1,000,000	6700 out of 1,000,000	3200 out of 1,000,000
CanSino Biologics	Severe drowsiness	Severe headache	Severe muscle pain	42,000 out of 1,000,000	54,000 out of 1,000,000	41,000 out of 1,000,000

	Benefits (protection)					
	Benefit 1	Benefit 2	Benefit 3	*P* (Benefit 1)	*P* (Benefit 2)	*P* (Benefit 3)
AstraZeneca	COVID-19 infection	Severe COVID-19	Death from COVID-19	78%	91%	92%
BioNTech/Pfizer	COVID-19 infection	Severe COVID-19	Death from COVID-19	95%	96%	98%
Moderna	COVID-19 infection	Severe COVID-19	Death from COVID-19	96%	97%	98%
Johnson & Johnson	COVID-19 infection	Severe COVID-19	Death from COVID-19	71%	86%	82%
Novavax	COVID-19 infection	Severe COVID-19	Death from COVID-19	90%	87%	–
Sinovac	COVID-19 infection	Severe COVID-19	Death from COVID-19	53%	71%	73%
Bharat Biotech	COVID-19 infection	Severe COVID-19	Death from COVID-19	78%	93%	–
CanSino Biologics	COVID-19 infection	Severe COVID-19	Death from COVID-19	58%	92%	–

Our specific inclusion criteria were as follows: We included all vaccines for which reliable clinical trial data of levels 3 and 4 were available in English at the time the study was designed. Initially, we also planned to include the Sputnik vaccine, but it was dropped when Russia launched the full-scale invasion of Ukraine.

### Vaccine evidence data

The vaccine evidence information (i.e., vaccine effectiveness, side effects, and the corresponding probabilities) about the eight vaccines that were presented to the participants in the mouse lab choice is provided in Table 1. A list of the data sources we used was sent to the participants after they had completed the study; the list can be obtained upon request. In general, we drew on phase 4 trials (i.e., data collected from monitoring the vaccine after releasing it to the public) for the data on vaccines’ effectiveness. If phase 4 trials were unavailable, we used results from phase 3 clinical trials (i.e., double-blind clinical trials involving thousands or tens of thousands of participants). The main sources for the side effects and their frequencies were also results from phase 3 trials or government reports. The latter were used mainly to obtain the frequencies of extremely rare side effects. We selected the three most severe side effects for each vaccine listed in the sources.

The eight selected vaccines included 15 side effects differing in severity: (1) Mild side effects: fever, tiredness, headache, and muscle pain; (2) Severe side effects: severe general discomfort, severe drowsiness, severe tiredness, severe headache, and severe muscle pain; (3) Extreme side effects: blood clots (thrombosis with thrombocytopenia syndrome), immune system attacking the nerves (Guillain-Barré syndrome) or the blood (immune thrombocytopenia), facial paralysis, heart muscle inflammation (myocarditis), and heart membrane inflammation (pericarditis).

### Experimental design

All participants explicitly consented to the conditions of the study. The study had four main parts: (1) a Mouselab task, (2) a willingness-to-pay task implemented for exploratory analyses and not reported here, (3) an affect rating task, and (4) a post-experimental survey. Each task began with a brief introduction of its general purpose. The procedure was programmed using JavaScript and JSpsych by the first author and Maik Messerschmidt from the research IT support team of the Center for Adaptive Rationality at the Max Planck Institute for Human Development. The procedure, including the consent form, is available in a preview mode at https://covid-vax.exp.arc.mpib.org/.

#### Mouselab task

Participants were presented with information on eight existing COVID-19 vaccines, one after the other, and were asked to indicate for each vaccine whether they would be willing to get vaccinated with it. The task started with a general introduction followed by detailed explanations of the task and the vaccine-related information, and a tutorial consisting of an example decision in which participants could try out how the information inspection boxes work. The vaccine-related information consisted of the developer/brand, country of origin, vaccine technology, risks (side effects and their frequencies), and benefits (effectiveness of the vaccine against COVID-19 infection, severe illness, and death due to the disease).

In each trial, the vaccine brand, country of origin, and vaccine technology were visible at the top of the screen. The outcomes of the vaccine (i.e., side effects and benefits) and their probabilities were hidden behind labeled black boxes; this information could be revealed by moving the mouse cursor over the box (Fig. 2). The information was visible as long as the cursor hovered over the box. Participants could freely explore the information as long and as often as they wished before making a decision, and the program recorded each hovering event. To approximate how vaccination risks and benefits tend to be presented in real life, the probabilities of side effects were presented as number of cases per 1,000,000 people, and effectiveness was presented using percentages that designated the relative risk reduction in the vaccinated population relative to the unvaccinated population. The presentation order of the vaccines, the relative position of risks to benefits, the relative positions of probabilities to outcomes, and the yes/no buttons were randomized between participants. However, to avoid confusion, the relative position of probabilities to outcomes and the yes/no buttons were held constant for each participant.

Before the actual task, participants were presented with a statement on the reliability of the presented data and were asked to “assume that the figures presented refer to the current wave of the pandemic and apply to you personally”: *Please keep in mind that the figures provided for the vaccines were taken from official sources (vaccine package leaflets, clinical trial reports, government reports) and reflect the best current state of knowledge. However, as the pandemic evolves, these figures may change, especially as new variants emerge. In addition, data may vary across countries, age groups, and health conditions, and due to other factors. It is therefore possible that the figures presented here deviate from those you may have encountered in other contexts. This is unavoidable, but it does not mean that the figures presented here are incorrect.* ***For the purpose of the study, please assume that the figures presented refer to the current wave of the pandemic and apply to you personally****.*

#### Affect rating task

This task consisted of two parts: affect ratings of the potential risks of the vaccines and affect ratings of the potential benefits. The presentation order of these parts was randomized between participants. Participants were asked to rate the overall negative and positive affect associated with each side effect and benefit.

The instruction for rating the side effects was as follows: *In this task, you will be presented with a list of possible side effects. Your task is to imagine experiencing each of them after a COVID-19 vaccination. Please indicate the amount of* ***negative emotion*** *you would feel as a result of experiencing the event. We mean any negative emotion, such as feeling distressed, upset, guilty, ashamed, hostile, irritated, nervous, jittery, scared, or afraid*.

Participants were then shown a Likert matrix table with 15 rows, each corresponding to one of the side effects (see Table 1).

The instruction for rating the vaccination benefits was as follows: *In this task, you will be presented with a list of the negative outcomes that vaccines protect against. Your task is to imagine that you are fully protected from each outcome. Please assess the amount of* ***positive emotion*** *you would feel as a result of being protected from the event. We mean any positive emotion, such as feeling excited, enthusiastic, proud, determined, relieved, strong, or active*.

Participants were then shown a Likert matrix table with three rows, each corresponding to one of the benefits (see Table 1).

The rating scale and example emotions listed in the instructions were based on the PANAS scale^54^. The labels for the negative and positive scales were identical. In both parts of this task, the order of the outcomes in the rating matrices was randomized for each participant.

#### Post-experimental survey

At the end of the study, we collected the following demographic information: sex (male, female, other), age in years (open-ended), racial identity (White, Black, Asian, multiracial, other), education (≤high school, some college education, Bachelor’s degree, ≥Master’s degree), political orientation (Democrat, Republican, Independent, other), and annual income (0–$30,000, $30,001–$60,000, $60,001–$99,999, ≥$100,000). Participants then reported the number of COVID-19 vaccinations they had received (open-ended), which vaccine they had received (BioNTech/Pfizer, Johnson & Johnson, Moderna), and how many times they had tested positive for COVID-19 (open-ended). We also asked: *In your opinion, how likely is it that in the future you will* (get COVID-19, get severe COVID-19, die from COVID-19). Participants answered this question using a rating scale with four options: definitely not, not likely, somewhat likely, and very likely. To measure vaccination attitude, we asked: *How would you characterize your general attitude towards vaccination against COVID-19?* (neutral, pro, against). The final question was open-ended: *Were there specific reasons for how you searched for information about the vaccines in the decision task? How would you characterize your search behavior?*

### Preprocessing of information inspection data

An instance of information inspection was defined as an event during which a participant hovered a mouse cursor over a labeled black box (Fig. 2a). Following standard practice, inspections that lasted less than 200 milliseconds were assumed to be incidental and removed from further analyses^55^. The remaining inspection data was used to construct trial-level (i.e., relating to a single vaccination decision) indices of deliberate ignorance for later usage in statistical and computational modeling. We based our analyses on the number of inspections of each piece of information rather than on total inspection times. The information we presented varied in format and character lengths (e.g., frequencies, percentages, text), which could affect inspection duration.

We distinguished between three levels of deliberate ignorance: full, partial, and none. The level of full deliberate ignorance was assigned to trials in the decision task in which no information on vaccine evidence was inspected. The level of partial deliberate ignorance was assigned to trials in which at least one information box on vaccine evidence was uncovered, excluding trials in which all information was inspected. The level of no deliberate ignorance was assigned to trials in which each piece of information on vaccine evidence was inspected at least once.

For all types of probability neglect investigated in the analyses (i.e., for benefits, side effects, and extreme, severe, and mild side effects), we distinguished between two levels: probability neglect either occurred or did not occur in the information inspection phase prior to the vaccination decision. A trial was classified as involving probability neglect of side effects if probability information for at least one side effect was not inspected, but the corresponding side effect was inspected; the same logic was used for benefits and for specific groups of side effects (i.e., mild, serve, or extreme).

### Statistical modeling

All statistical models presented were estimated using the brms package^56^ called from R^57^. All predictors were categorical and always coded with sum-to-zero contrasts. Posterior distributions of the models were estimated using four chains. Each chain consisted of 4000 iterations. The first half was used for burn-in, and only every second sample was recorded from the second half, resulting in 4000 recorded samples in total. The sampling procedure resulted in well-mixed chains, as indicated by $\hat{R}$ values lower than 1.01.

We ran Bayesian hierarchical logistic regressions with a random intercept across participants for binary outcome variables (i.e., vaccine acceptance and probability neglect). For ordinal outcome variables (i.e., deliberate ignorance and affect ratings), we used Bayesian hierarchical ordinal regressions, developed specifically for these types of variables^58^. As priors for the regression coefficients in both types of models, we used zero-centered Student’s t-distribution, with a scale parameter of 2.5 and 3 degrees of freedom, which is considered a weakly informative prior (see: https://github.com/stan-dev/stan/wiki/Prior-Choice-Recommendations).

The models were able to adequately capture the patterns in the data, as indicated by posterior predictive checks^59^. The approximated out-of-sample predictive performance of the reported statistical models varied from. 7 to. 87 (see Supplementary Information for more details on the evaluation of statistical models).

#### Predicted outcome values and pairwise comparisons

The posterior predicted outcome values presented in Figs. 3–6 were calculated using the conditional_effects function from the brms package used to estimate the models^56^. The predictions for a given predictor from a regression with multiple predictors were derived by setting all other predictor values to zero. Because all our models contained categorical predictors coded with sum-to-zero contrasts, these predictions are equivalent to taking the posterior of the global intercept from the model and adding it to the posteriors of the regression weights of a predictor of interest and passing the resulting values through the relevant link function (e.g., the inverse-logit function in the case of logistic regression).

To compute evidence for a difference between any two levels of a categorical predictor (reversed blue triangles in Figs. 4–6), we also drew on the posterior distributions of the regression weights. For a predictor with only two levels (e.g., attentional probability neglect: yes vs. no), we inferred that the data provided evidence for a difference in the outcome variable if the 95% HDI of the posterior distribution of the regression weight excluded zero.

For categorical predictors with more than two levels, the procedure for pairwise comparisons was more complex due to the sum-to-zero contrast factor coding used to estimate the models. First, for each factor level (e.g., vaccination attitude groups), the posterior predicted outcome value on the scale of the linear predictor was computed using the respective factor coding scheme and regression weights. Second, these posterior predicted outcome values were subtracted from each other to derive the posterior distribution of the outcome difference between any two levels of a factor of interest (e.g., anti-vaccination and neutral attitudes). Again, we inferred that the data provided evidence for a difference in the outcome variable between the two levels of a factor if the 95% HDI of the posterior distribution of the regression weight excluded zero.

### Computational modeling

The model was written in the Stan programming language for statistical computing^60^. The posterior distribution of the model parameters was estimated using the rstan package^61^ called from R^57^. The sampling procedure from the posterior distribution was based on four chains, each consisting of 2000 warm-up and 3000 subsequent samples. Every other sample was recorded, providing 6000 recorded samples in total. The sampling procedure resulted in autocorrelation-free and well-mixed chains, as indicated by $\hat{R}$ values lower than 1.01.

The leave-one-out balanced predictive accuracy for the models reported in the main text was 0.81, 0.74, and 0.76 for the anti-vaccination, neutral, and pro-vaccination groups, respectively. The Supplementary Information provides additional analyses of model performance evaluation, including a comparison of the performance of alternative, simpler models.

#### Formal model specification

The probability of individual *i* accepting the vaccine *v*, denoted by *P*(accept), was given by

2 $$P(\,\text{accept}\,)\,=\frac{1}{1+\exp -({\beta }_{i}+{\sum }_{j}{X}_{v}{\beta }_{j}+\varphi {V}_{i,v})}.$$

The *β*_*i*_ parameter represents an individual-level decision bias: It indicates to what extent the participant *i* tends to accept or refuse a vaccine irrespective of the vaccine properties and evidence. The term *X*_*v*_*β*_*j*_ consists of a 3 × 4 matrix of sum-to-zero contrasts *X*_*v*_ and corresponding *β*_*j*_ parameters. The first two columns of *X*_*v*_ code the country of origin (United States, China, other); the next two columns code the vaccine technology (mRNA, vector, other). The term *ϕV*_*i*,*v*_ is the participant’s *i* subjective value of vaccine *v* determined based on prospect theory^32^. The *V*_*i*,*v*_ component consists of the value function *v*(*a*), which takes participant *i*’s affect ratings for side effects *a*_*i*,*se*_ and benefits *a*_*i*,*b*_ as inputs, and of a probability weighting function *w*(*p*), which takes the probabilities of side effects *p*_*se*_ and benefits *p*_*b*_ as inputs (with the latter technically being the effectiveness of the vaccine, see Supplementary Information):

3 $${V}_{i,v}=\sum _{se}v({a}_{i,se})w({p}_{se})+\sum _{b}v({a}_{i,b})w({p}_{b}).$$

The value function *v*(*a*) has three cases, depending on whether the affect rating pertains to a side effect (*a*_*i*,*se*_), a benefit (*a*_*i*,*b*_), or an outcome ignored in the information inspection pre-decision phase:

4 $$v(a)=\left\{\begin{array}{ll}-{\lambda }_{i}| a{| }^{\alpha },\quad &\,\text{for}\,a={a}_{i,se}\text{}\\ (1-{\lambda }_{i}){a}^{\alpha },\quad &\,\text{for}\,a={a}_{i,b}\text{}\\ 0,\quad &\,{\rm{for}}\,{\rm{ignored}}\,{\rm{outcomes}}\,,\end{array}\right.$$

where the *λ*_*i*_ ∈ [0, 1] parameter is a measure of loss aversion estimated separately for each participant *i*. With *λ*_*i*_ = 0.5, both side effects and benefits are weighted equally, while values of *λ*_*i*_ > 0.5 indicate an overweighting of side effects relative to benefits—which can be interpreted as loss aversion. Note that in most applications to monetary lotteries, the loss aversion parameter *Λ* is used as a multiplier of the negative consequences and estimated on the scale of positive real numbers. Our approach is algebraically equivalent since *Λ* = *λ*_*i*_/(1 − *λ*_*i*_), but resulted in better model convergence.

The *α* > 0 parameter allows for a nonlinear transformation of the affect ratings. This may be necessary because the affect ratings were measured using an ordinal Likert scale (see Fig. 2b), and the assumption of equal distances between the scale levels may not hold here (but the model allows for a linear mapping with *α* = 1). Note that when the *α* parameter is large, for example, when *α* = 3, the value of an extreme affect coded as *a* = 5 would be *v*(5) = *λ* × 125. Such large values would have a dominating effect within the logit function in Equation (2). For this reason, the *V*_*i*,*v*_ component is scaled with the *φ* ∈ [0, 1] parameter—allowing any level of nonlinearity in the affect ratings scale but ensuring that the final value of the *φV*_*i*,*v*_ term is as large as supported by the data.

The function *w*(*p*) in Equation (2) transformed the probabilities of side effects and benefits into decision weights and had two cases, depending on whether the probability was inspected or neglected (i.e., deliberately ignored):

5 $$w(p)=\left\{\begin{array}{ll}\exp (-{(-\ln p)}^{{\gamma }_{i}})\quad &\,{\text{if}}\,\,p\,\,{\rm{was}}\,{\rm{inspected}}\,\\ 0.5\quad &\,{\text{if}}\,\,p\,\,{\rm{was}}\,{\rm{neglected}}\,.\end{array}\right.$$

The first case in Equation (5) is an inverse S-shaped probability weighting function^62^ that transforms objective probabilities into subjective decision weights. The free parameter *γ*_*i*_ ∈ [0, 1] governs the curvature of the probability weighting function and is interpreted as probability sensitivity, with higher values indicating higher sensitivity. When *γ*_*i*_ = 0, the function becomes a horizontal line with all decision weights *w*(*p*) = 0.37. When *γ*_*i*_ = 1, the function indicates perfect probability sensitivity, that is, *w*(*p*) = *p*. The second case of Equation (5) applies in situations in which an outcome was inspected but its corresponding probability was not; in the main analyses, for these instances of probability neglect, we set *w*(*p*) = 0.5, which means that the decision-maker acknowledges the probabilistic nature of the inspected outcome. See the Supplementary Information for an extended discussion on the assumptions underlying the estimation of the weighting function, including the value of the neglected probability and interpretation of the vaccine effectiveness.

In sum, three parameters of the model were estimated on the individual level (i.e., for each participant): decision bias *β*_*i*_, loss aversion *λ*_*i*_, and probability sensitivity *γ*_*i*_. The parameters *α* and *φ* were only estimated on the group level because there were only eight data points (i.e., decisions) per participant, and we had no theoretical interest in estimating these parameters on the individual level.

#### Stan implementation and prior distributions

The individual-level parameters were modeled as a sum of a corresponding group-level parameter and individual-level displacements *ζ*_*i*_:

6 $$\begin{array}{c}{\beta }_{i}=\beta +{\zeta }_{i}^{\beta }\\ {\lambda }_{i}=\Phi ({\lambda }^{\Phi }+{\zeta }_{i}^{\lambda })\\ {\gamma }_{i}=\Phi ({\gamma }^{\Phi }+{\zeta }_{i}^{\gamma }),\end{array}$$

where the function Φ() is an approximation to the cumulative normal distribution function implemented in Stan and ensures that the resulting individual-level parameters are always in the required 0–1 range. The individual displacements are assumed to follow a multivariate normal distribution with mean *μ* = [0, 0, 0] and variance–covariance matrix *Σ*, also estimated from the data.

In terms of priors, we used standard normal distribution for the individual- and group-level decision biases *β*_*i*_ and *β*, respectively, and also for the vaccine effects coefficients *β*_*v*_, thus assuming that the biases to accept or refuse a vaccine are equally likely.

We could also use the standard normal distribution as the priors for the group-level parameters on the probit scale: *λ*^Φ^, *γ*^Φ^, and *φ*^Φ^, which after transformation to the scale of actual parameter values via probit function Φ^−1^() resulted in uniform priors on the 0–1 range—in line with the theoretical bounds of the parameters:

7 $$\begin{array}{c}\varphi ={\Phi }^{-1}({\varphi }^{\Phi })\\ \lambda ={\Phi }^{-1}({\lambda }^{\Phi })\\ \gamma ={\Phi }^{-1}({\gamma }^{\Phi }).\\ \end{array}$$

The *α* parameter was also modeled on the scale of real values and received a normal prior with a mean of zero and a standard deviation of 0.5. The parameter was then transformed via the exponential function to the scale of positive reals, resulting in a prior with a mode of one (i.e., linear usage of the Likert scale) and assuming that the plausible parameter values are in the 0–4 range.

Finally, to model the multivariate distribution of the individual displacements *ζ*_*i*_, we used weakly informative Lewandowski-Kurowicka-Joe (LKJ) prior with parameter *η* = 5 for the correlation matrix, which assumed that the most probable correlations between the individual parameters were in the range from −0.5 to 0.5. The prior for the standard deviation of the individual displacements ${\zeta }_{i}^{\beta }$ was the gamma distribution with shape and rates equal to 2 and 1, respectively, thus ensuring that the parameter is positive and likely in the 0–3 range. The priors for the standard deviations of the individual displacements ${\zeta }_{i}^{\lambda }$ and ${\zeta }_{i}^{\gamma }$ were normal distributions with a mean of 0.5 and standard deviation of 0.13, ensuring that the resulting standard deviation is within 0–1 range. This condition was necessary to avoid bimodal individual-level posterior distributions of the *λ*_*i*_ and *γ*_*i*_ parameters after the Φ() transformation in Equation (6).

### Preregistration

The study design, including sample size and the number of vaccination decisions, was preregistered on April 19, 2022: https://aspredicted.org/66W_95Q. Four research questions were formulated, all relating to probability neglect. Specifically, we were interested in (1) whether people exhibit probability neglect in vaccination decisions, (2) whether probability neglect rates are associated with vaccination attitudes, (3) how probability neglect is associated with vaccination decisions; (4) to what extent probability neglect applies to side effects and benefits. All these research questions are addressed in the main text in the section “How was probability ignorance related to vaccine refusal?” using analytical methods specified in the preregistration. In the preregistration, we also considered weaker definitions of probability neglect (e.g., a lower number of acquisitions of probability information than of the corresponding outcome) than the one used in the paper. However, we decided to use the stricter definition of probability neglect here: acquisition of outcome but not probability information.

At the end of the preregistration, in the section titled *Other*, we also mentioned our plan to analyze the data using computational modeling and noted that to this end, we would collect affect ratings for side effects and benefits to use as numerical inputs in the models. Our modeling approach followed current best practices (see “Methods: Computational modeling”) and was based on prospect theory—a model previously used to analyze medical choices^25–29,63^. The model we developed deviated from the standard applications to simple monetary lotteries because vaccination decisions are more complex. Our model was developed to accurately capture patterns in the data and account for various factors driving vaccination decisions (see Supplementary Information).

## Supplementary information

Supplementary Information

## Data Availability

All study data are available on the first author’s public GitHub repository: https://github.com/kfulawka/vax_info_neglect.

## Code availability

The code needed to reproduce the analyses reported in the article is available on the first author’s public GitHub repository: https://github.com/kfulawka/vax_info_neglect.

## References

[1] World Health Organization. *Ten Threats to Global Health in 2019*. https://www.who.int/news-room/spotlight/ten-threats-to-global-health-in-2019 (2019).

[2] Dubé, E. et al. Vaccine hesitancy: an overview. *Hum. Vaccin. Immunother.* **9**, 1763–1773 (2013). 23584253 10.4161/hv.24657 PMC3906279

[3] Aw, J., Seng, J. J. B., Seah, S. S. Y. & Low, L. L. COVID-19 vaccine hesitancy—a scoping review of literature in high-income countries. *Vaccines* **9**, 900 (2021). 34452026 10.3390/vaccines9080900 PMC8402587

[4] Solís Arce, J. S. et al. COVID-19 vaccine acceptance and hesitancy in low- and middle-income countries. *Nat. Med.* **27**, 1385–1394 (2021). 34272499 10.1038/s41591-021-01454-y PMC8363502

[5] World Health Organization. *Vaccination and Trust: How Concerns Arise and the Role of Communication in Mitigating Crises*. https://apps.who.int/iris/handle/10665/343299 (2017).

[6] Lewandowsky, S. et al. *The COVID-19 Vaccine Communication Handbook. A Practical Guide for Improving Vaccine Communication and Fighting Misinformation*. https://www.movementdisorders.org/MDS-Files1/The_COVID-19_Vaccine_Communication_Handbook.pdf (2022).

[7] Brick, C., McDowell, M. & Freeman, A. L. Risk communication in tables versus text: a registered report randomized trial on ‘fact boxes’. *R. Soc. Open Sci.* **7**, 190876 (2020). 32269779 10.1098/rsos.190876 PMC7137953

[8] Charles, N. *Suspicion: Vaccines, Hesitancy, and the Affective Politics of Protection in Barbados* (Duke Univ. Press, 2021).

[9] Hausman, B. L. *Anti/Vax: Reframing the Vaccination Controversy* (ILR Press, 2019).

[10] Kreps, S., Dasgupta, N., Brownstein, J. S., Hswen, Y. & Kriner, D. L. Public attitudes toward COVID-19 vaccination: the role of vaccine attributes, incentives, and misinformation. *NPJ Vaccines* **6**, 73 (2021). 33990614 10.1038/s41541-021-00335-2 PMC8121853

[11] Stöckli, S. et al. Which vaccine attributes foster vaccine uptake? a cross-country conjoint experiment. *PLoS ONE* **17**, e0266003 (2022). 35507554 10.1371/journal.pone.0266003 PMC9067644

[12] Freeman, D. et al. COVID-19 vaccine hesitancy in the UK: the Oxford coronavirus explanations, attitudes, and narratives survey (OCEANS) II. *Psychol. Med.* **52**, 3127–3141 (2022). 33305716 10.1017/S0033291720005188 PMC7804077

[13] Marzo, R. R. et al. Perceived COVID-19 vaccine effectiveness, acceptance, and drivers of vaccination decision-making among the general adult population: a global survey of 20 countries. *PLoS Negl. Trop. Dis.* **16**, e0010103 (2022). 35089917 10.1371/journal.pntd.0010103 PMC8797205

[14] Prosser, L. A. et al. A discrete choice analysis comparing COVID-19 vaccination decisions for children and adults. *JAMA Netw. Open* **6**, e2253582 (2023). 36716030 10.1001/jamanetworkopen.2022.53582 PMC9887501

[15] Cerda, A. A. & García, L. Y. Hesitation and refusal factors in individuals’ decision-making processes regarding a coronavirus disease 2019 vaccination. *Front. Public Health* **9**, 626852 (2021). 33968880 10.3389/fpubh.2021.626852 PMC8096991

[16] Kaplan, R. M. & Milstein, A. Influence of a COVID-19 vaccine’s effectiveness and safety profile on vaccination acceptance. *Proc. Natl Acad. Sci. USA* **118**, e2021726118 (2021). 33619178 10.1073/pnas.2021726118 PMC7958192

[17] Liu, H. et al. How information processing and risk/benefit perception affect COVID-19 vaccination intention of users in online health communities. *Front. Public Health* **11**, 1043485 (2023). 36895686 10.3389/fpubh.2023.1043485 PMC9989022

[18] Strickland, J. C. et al. Behavioral economic methods to inform infectious disease response: prevention, testing, and vaccination in the COVID-19 pandemic. *PLoS ONE* **17**, e0258828 (2022). 35045071 10.1371/journal.pone.0258828 PMC8769299

[19] Toro-Ascuy, D. et al. Factors influencing the acceptance of COVID-19 vaccines in a country with a high vaccination rate. *Vaccines* **10**, 681 (2022). 35632437 10.3390/vaccines10050681 PMC9145438

[20] Hertwig, R. & Engel, C. Homo ignorans: deliberately choosing not to know. *Perspect. Psychol. Sci.* **11**, 359–372 (2016). 27217249 10.1177/1745691616635594

[21] Jennings, W. et al. Lack of trust, conspiracy beliefs, and social media use predict COVID-19 vaccine hesitancy. *Vaccines* **9**, 593 (2021). 34204971 10.3390/vaccines9060593 PMC8226842

[22] Robertson, E. et al. Predictors of COVID-19 vaccine hesitancy in the UK household longitudinal study. *Brain Behav. Immun.* **94**, 41–50 (2021). 33713824 10.1016/j.bbi.2021.03.008 PMC7946541

[23] Sunstein, C. R. Probability neglect: emotions, worst cases, and law. *Yale Law J.* **112**, 61–107 (2002). 10.2307/1562234

[24] Sunstein, C. R. & Zeckhauser, R. Overreaction to fearsome risks. *Environ. Resour. Econ.* **48**, 435–449 (2011). 10.1007/s10640-010-9449-3

[25] Pachur, T., Hertwig, R. & Wolkewitz, R. The affect gap in risky choice: affect-rich outcomes attenuate attention to probability information. *Decision* **1**, 64–78 (2014). 10.1037/dec0000006

[26] Pachur, T., Suter, R. S. & Hertwig, R. How the twain can meet: prospect theory and models of heuristics in risky choice. *Cogn. Psychol.* **93**, 44–73 (2017). 28189037 10.1016/j.cogpsych.2017.01.001

[27] Suter, R. S., Pachur, T., Hertwig, R., Endestad, T. & Biele, G. The neural basis of risky choice with affective outcomes. *PLoS ONE* **10**, e0122475 (2015). 25830918 10.1371/journal.pone.0122475 PMC4382171

[28] Lejarraga, T., Pachur, T., Frey, R. & Hertwig, R. Decisions from experience: from monetary to medical gambles. *J. Behav. Decis. Mak.* **29**, 67–77 (2016). 10.1002/bdm.1877

[29] Suter, R. S., Pachur, T. & Hertwig, R. How affect shapes risky choice: distorted probability weighting versus probability neglect. *J. Behav. Decis. Mak.* **29**, 437–449 (2016). 10.1002/bdm.1888

[30] Mousavi, S. & Gigerenzer, G. Risk, uncertainty, and heuristics. *J. Bus. Res.* **67**, 1671–1678 (2014). 10.1016/j.jbusres.2014.02.013

[31] Reyna, V. F., Broniatowski, D. A. & Edelson, S. M. Viruses, vaccines, and COVID-19: explaining and improving risky decision-making. *J. Appl. Res. Mem. Cogn.* **10**, 491–509 (2021). 34926135 10.1016/j.jarmac.2021.08.004 PMC8668030

[32] Tversky, A. & Kahneman, D. Advances in prospect theory: cumulative representation of uncertainty. *J. Risk Uncertain.* **5**, 297–323 (1992). 10.1007/BF00122574

[33] Waters, E. A., Weinstein, N. D., Colditz, G. A. & Emmons, K. M. Aversion to side effects in preventive medical treatment decisions. *Br. J. Health Psychol.* **12**, 383–401 (2007). 17640453 10.1348/135910706X115209

[34] Waters, E. A., Weinstein, N. D., Colditz, G. A. & Emmons, K. M. Reducing aversion to side effects in preventive medical treatment decisions. *J. Exp. Psychol. Appl.* **13**, 11 (2007). 17385998 10.1037/1076-898X.13.1.11

[35] Waters, E. A., Weinstein, N. D., Colditz, G. A. & Emmons, K. Explanations for side effect aversion in preventive medical treatment decisions. *Health Psychol.* **28**, 201 (2009). 19290712 10.1037/a0013608 PMC2657933

[36] Loewenstein, G. Hot–cold empathy gaps and medical decision making. *Health Psychol.* **24**, S49–S56 (2005). 16045419 10.1037/0278-6133.24.4.S49

[37] Izadi, S., Pachur, T., Wheeler, C., McGuire, J. & Waters, E. A. Spontaneous mental associations with the words “side effect”: implications for informed and shared decision making. *Patient Educ. Couns.* **100**, 1928–1933 (2017). 28583721 10.1016/j.pec.2017.05.029 PMC5573624

[38] Waters, E. A., Pachur, T. & Colditz, G. A. Side effect perceptions and their impact on treatment decisions in women. *Med. Decis. Mak.* **37**, 193–203 (2017). 10.1177/0272989X16650664 PMC5121093 27216581

[39] Payne, J. W., Bettman, J. R. & Johnson, E. J. *The Adaptive Decision Maker* (Cambridge Univ. Press, 1993).

[40] Fuławka, K. & Pachur, T. An affective probability weighting function for risky choice with nonmonetary outcomes. In *Proc. Annual Meeting of the Cognitive Science Society*, Vol. 44 (eds Culbertson, J. et al.) 1025–1032. https://escholarship.org/uc/item/5bg7f816 (2022).

[41] Blastland, M., Freeman, A. L., van der Linden, S., Marteau, T. M. & Spiegelhalter, D. Five rules for evidence communication. *Nature* **587**, 362–364 (2020). 33208954 10.1038/d41586-020-03189-1

[42] Lindholt, M. F., Jørgensen, F., Bor, A. & Petersen, M. B. Public acceptance of COVID-19 vaccines: cross-national evidence on levels and individual-level predictors using observational data. *BMJ Open* **11**, e048172 (2021). 34130963 10.1136/bmjopen-2020-048172 PMC8210695

[43] Viskupič, F., Wiltse, D. L. & Meyer, B. A. Trust in physicians and trust in government predict COVID-19 vaccine uptake. *Soc. Sci. Q.* **103**, 509–520 (2022). 35600052 10.1111/ssqu.13147 PMC9115527

[44] Johnson, N. F. et al. The online competition between pro-and anti-vaccination views. *Nature* **582**, 230–233 (2020). 32499650 10.1038/s41586-020-2281-1

[45] Betsch, C. et al. A call for immediate action to increase COVID-19 vaccination uptake to prepare for the third pandemic winter. *Nat. Commun.* **13**, 7511 (2022). 36473855 10.1038/s41467-022-34995-y PMC9726862

[46] Wegwarth, O., Wagner, G. G., Spies, C. & Hertwig, R. Assessment of German public attitudes toward health communications with varying degrees of scientific uncertainty regarding COVID-19. *JAMA Netw. Open* **3**, e2032335 (2020). 33301021 10.1001/jamanetworkopen.2020.32335 PMC7729432

[47] Petersen, M. B., Bor, A., Jørgensen, F. & Lindholt, M. F. Transparent communication about negative features of COVID-19 vaccines decreases acceptance but increases trust. *Proc. Natl Acad. Sci. USA* **118**, e2024597118 (2021). 34292869 10.1073/pnas.2024597118 PMC8307373

[48] Wegwarth, O. et al. Vaccination intention following receipt of vaccine information through interactive simulation vs text among COVID-19 vaccine-hesitant adults during the omicron wave in Germany. *JAMA Netw. Open* **6**, e2256208 (2023). 36795411 10.1001/jamanetworkopen.2022.56208 PMC9936332

[49] Wang, K. et al. A multi-country test of brief reappraisal interventions on emotions during the COVID-19 pandemic. *Nat. Hum. Behav.* **5**, 1089–1110 (2021). 34341554 10.1038/s41562-021-01173-x PMC8742248

[50] Douglas, B. D., Ewell, P. J. & Brauer, M. Data quality in online human-subjects research: comparisons between MTurk, Prolific, CloudResearch, Qualtrics, and SONA. *PLoS ONE* **18**, e0279720 (2023). 36917576 10.1371/journal.pone.0279720 PMC10013894

[51] Peer, E., Rothschild, D., Gordon, A., Evernden, Z. & Damer, E. Data quality of platforms and panels for online behavioral research. *Behav. Res. Methods* **54**, 1643–1662 (2022). 10.3758/s13428-021-01694-3 PMC8480459 34590289

[52] Kunda, Z. The case for motivated reasoning. *Psychol. Bull.* **108**, 480 (1990). 2270237 10.1037/0033-2909.108.3.480

[53] Hertwig, R. & Engel, C. *Deliberate Ignorance: Choosing Not to Know*, Vol. 29 (MIT Press, 2021).

[54] Watson, D., Clark, L. A. & Tellegen, A. Development and validation of brief measures of positive and negative affect: the PANAS scales. *J. Pers. Soc. Psychol.* **54**, 1063–1070 (1988). 3397865 10.1037/0022-3514.54.6.1063

[55] Willemsen, M. C. & Johnson, E. J. *Visiting the Decision Factory: Observing Cognition with MouselabWEB and Other Information Acquisition Methods*, 21–42 (Psychology Press, 2011).

[56] Bürkner, P.-C. brms: an R package for Bayesian multilevel models using stan. *J. Stat. Softw.* **80**, 1–28 (2017). 10.18637/jss.v080.i01

[57] R Core Team. R: A Language and Environment for Statistical Computing. https://www.R-project.org/ (R Foundation for Statistical Computing, 2021).

[58] Bürkner, P.-C. & Vuorre, M. Ordinal regression models in psychology: a tutorial. *Adv. Methods Pract. Psychol. Sci.* **2**, 77–101 (2019). 10.1177/2515245918823199

[59] Gabry, J., Simpson, D., Vehtari, A., Betancourt, M. & Gelman, A. Visualization in Bayesian workflow. *J. R. Stat. Soc. A Stat. Soc.* **182**, 389–402 (2019). 10.1111/rssa.12378

[60] Carpenter, B. et al. Stan: a probabilistic programming language. *J. Stat. Softw.* **76**, 1–32 (2017). 36568334 10.18637/jss.v076.i01 PMC9788645

[61] Stan Development Team. RStan: The R Interface to Stan. R package version 2.21.7. https://mc-stan.org/ (2022).

[62] Prelec, D. The probability weighting function. *Econometrica* **66**, 497–527 (1998). 10.2307/2998573

[63] Van Houtven, G., Johnson, F. R., Kilambi, V. & Hauber, A. B. Eliciting benefit–risk preferences and probability-weighted utility using choice-format conjoint analysis. *Med. Decis. Mak.* **31**, 469–480 (2011). 10.1177/0272989X10386116 21310854

