# Supplementary material for: COVID-19 vaccine refusal is driven by deliberate ignorance and cognitive distortions
Source: NPJ Vaccines. 2024 Sep 14;9:167. doi: 10.1038/s41541-024-00951-8 (PMC11399437; doi:10.1038/s41541-024-00951-8)
Supplement: Supplementary file 1 — Supplementary Information [file 41541_2024_951_MOESM1_ESM.pdf]

COVID-19 Vaccine Refusal Is Driven by Deliberate Ignorance and Cognitive Distortions—Supplementary Information

Kamil Fuławka[1*], Ralph Hertwig[1], and Thorsten Pachur[1,2]

[1]Center for Adaptive Rationality, Max Planck Institute for Human Development
[2]School of Management, Technical University of Munich

* Corresponding author: fulawka@mpib-berlin.mpg.de

Distributions of Demographic Variables and Vaccination Attitudes

Figure 1 shows the distribution of the demographic variables in the study and the univariate relationships with the vaccination attitude.

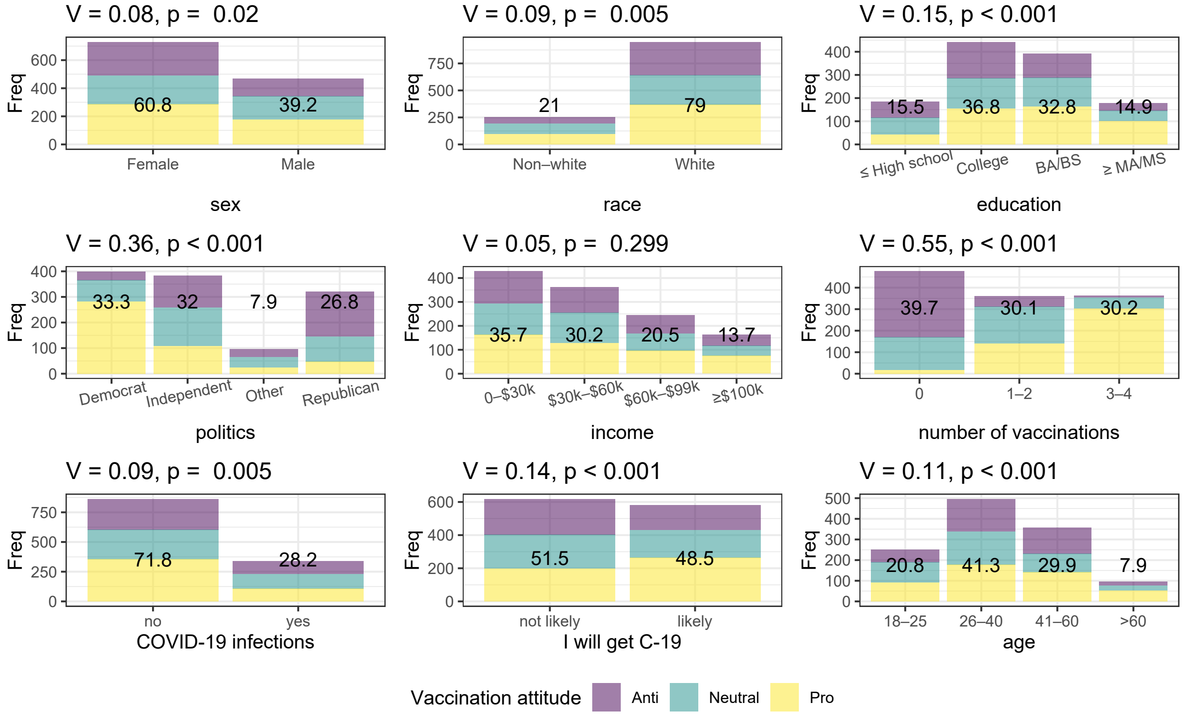

Supplementary Figure 1: The distribution of recorded demographic variables and relationships between the variables and vaccination attitudes. The black numbers are percentages of participants in a given category. The statistic in the panel's title is Cramér's V.

Statistical Analysis

Model Evaluation Analyses

We used posterior predictive checks (PPC) to evaluate the fit of a statistical model to the data—that is, to test how well a model captures the patterns in the data [1]. The PPC procedure consists of simulating the outcome variable using the predictors used to fit the model and the parameters of the fitted model. The distribution of the simulated outcome variable is then compared with the distribution of the outcome variable in the data. In general, the simulated and the empirical distributions aligned well, suggesting adequate model fit.

As the PPC procedure uses the same data twice (i.e., in model fitting and in generating the simulated outcome distribution), it should not be used to assess the predictive model performance [1]. To evaluate a model's out-of-sample predictive performance, we used the leave-one-out cross-validation method, which allowed us to evaluate how well the model is expected to predict future data while controlling for model complexity. Specifically, we used the approximated leave-one-out expected log pointwise predictive density statistic ($\mathrm{elpd}_{\mathrm{loo}}$) from the loo package [2]. In the context of the outcome variables analyzed here (i.e., binary and ordinal outcomes), the $\mathrm{elpd}_{\mathrm{loo}}$ statistic represented the sum, over all data points, of log-likelihoods (i.e., the predicted probability of the observed outcome value) for the ith observation, estimated from a model fitted to the data set excluding the ith observation. The exact value of the $\mathrm{elpd}_{\mathrm{loo}}$ statistic depends on the number of data points and should be interpreted in this context.

For our purpose, which was to ensure that the models used for inference could be assumed to represent the data well, we focused on checking whether the models with predictors predicted future data better than the corresponding *null* models, that is, models without predictors and consisting only of individual intercepts. To this end, we calculated $\mathrm{elpd}_{\mathrm{loo}}$ for the null models and the models reported in the article. All models reported exhibited better out-of-sample predictive performance than the null models (see Figure 2).

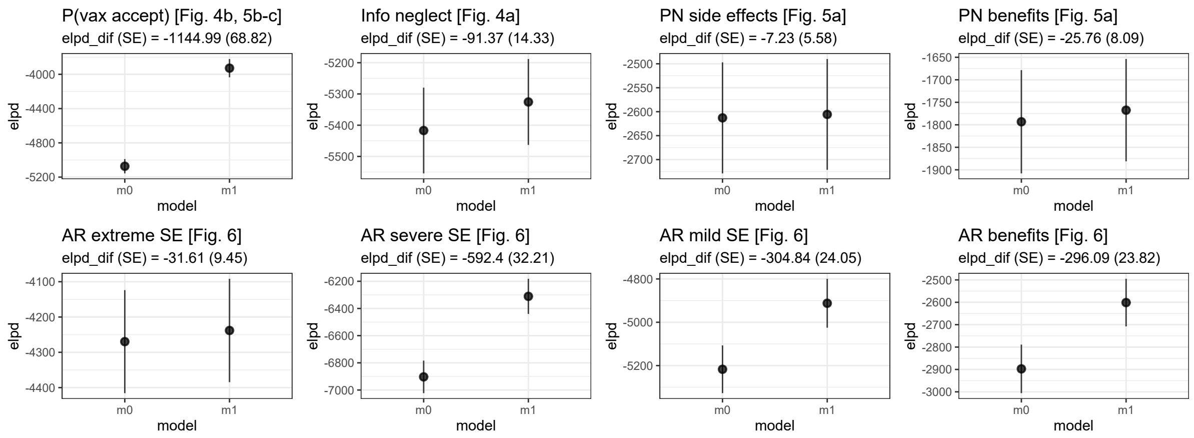

Supplementary Figure 2: Comparison of out-of-sample predictive performance of full statistical models (m1—presented in the main text) against null models (m0). Panel titles refer to the figures in the main text. See the paragraphs above for details.

We also computed leave-one-out balanced accuracy ($\mathrm{ba}_{\mathrm{loo}}$) to obtain a more intuitive measure of the predictive performance of the reported models. Balanced accuracy is the average of the correct model predictions across

all outcome variable levels. The ba_{loo} values for the statistical models reported in the article were ba_{loo} = 0.8 for the Bayesian hierarchical logistic regression model with vaccine acceptance as the outcome variable and all predictors as in Figure 5c (i.e., all demographic and individual factors, and information and attentional probability neglect indices); ba_{loo} = .82 for the Bayesian hierarchical ordinal regression with deliberate ignorance as the outcome variable and demographic and individual factors as predictors (Fig. 4a); ba_{loo} = 0.78 and ba_{loo} = 0.7 for the Bayesian hierarchical logistic regressions with probability neglect for benefits and side effects, respectively, as outcome variables and with vaccination attitude and vaccine brand as predictors (Fig. 5a; due to a relatively low proportion of target events, we used prediction cutoffs that minimized the differences in accuracies between predicting no-neglect and neglect occurrence events); ba_{loo} = .77, ba_{loo} = .84, ba_{loo} = .83, and ba_{loo} = .87 for the Bayesian hierarchical ordinal regressions with affect ratings for extreme, severe, and mild side effects, and for benefits, respectively, with vaccination attitudes and outcomes from the respective groups as predictors (Fig. 6).

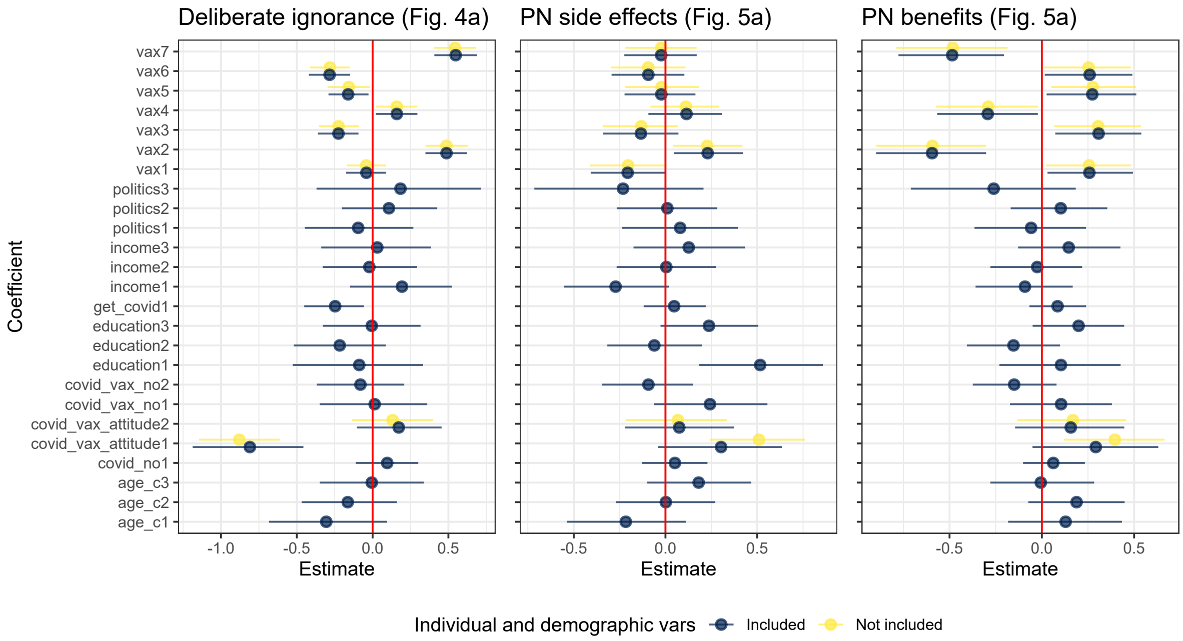

Supplementary Figure 3: Regression weights with 95% HDI for two sets of statistical models. Dark blue shows weights from a model including all demographic variables, vaccine brand, and vaccination attitudes. Yellow shows weights for models with the same outcome variables but includes only vaccine brand and vaccination attitudes as predictors. Panel titles correspond to figures in the main text

Model Specification Analyses

We conducted a series of model specification analyses to assess the robustness of the main effects reported in the main text against the specification of the regression models with various sets of predictors.

First, we checked whether the observed differences between the attitude groups in the proportion of decisions with various types of deliberate ignorance (Figures 4a and 5a in the main text) depend on including demographic variables in the regression model. To this end, we estimated two regression models with differing sets of predictors: (1) a model containing only the fixed effects of the attitude group and vaccine brand and (2) a model additionally containing all demographic factors (see Figure 3 in the main text). For all three outcome variables, the estimated

regression coefficients for the effects of vaccination attitudes were qualitatively the same when estimated with and without the demographic factors (Fig. 3). However, for both types of probability neglect, the 95% HDI of the regression weight *covid_vax_attitude1* includes zero in the regression model with demographics. This indicates that the differences in proportions of probability neglect between attitude groups are at least partially linked to other individual and demographic factors.

Second, we tested if the relationships between vaccination decisions and deliberate ignorance (including probability neglect types; Fig. 4b and Fig. 5b) depend on the regression model specification. The effects of probability neglect of vaccine benefits and side effects reported in the main text were estimated in separate models, including the effects of vaccination attitude and vaccine brand, and their interaction. However, when estimated in a single model, these effects cancel each other due to high intercorrelation. In the next step, we therefore focused on the effects of the four types of probability neglect presented in Figure 5b (i.e., separate indices for extreme, severe, and mild side effects, and for benefits). These effects remained qualitatively the same when estimated simultaneously within a model including only vaccination attitude and vaccine brand, and a model with all other demographic and individual predictors (Fig. 4).

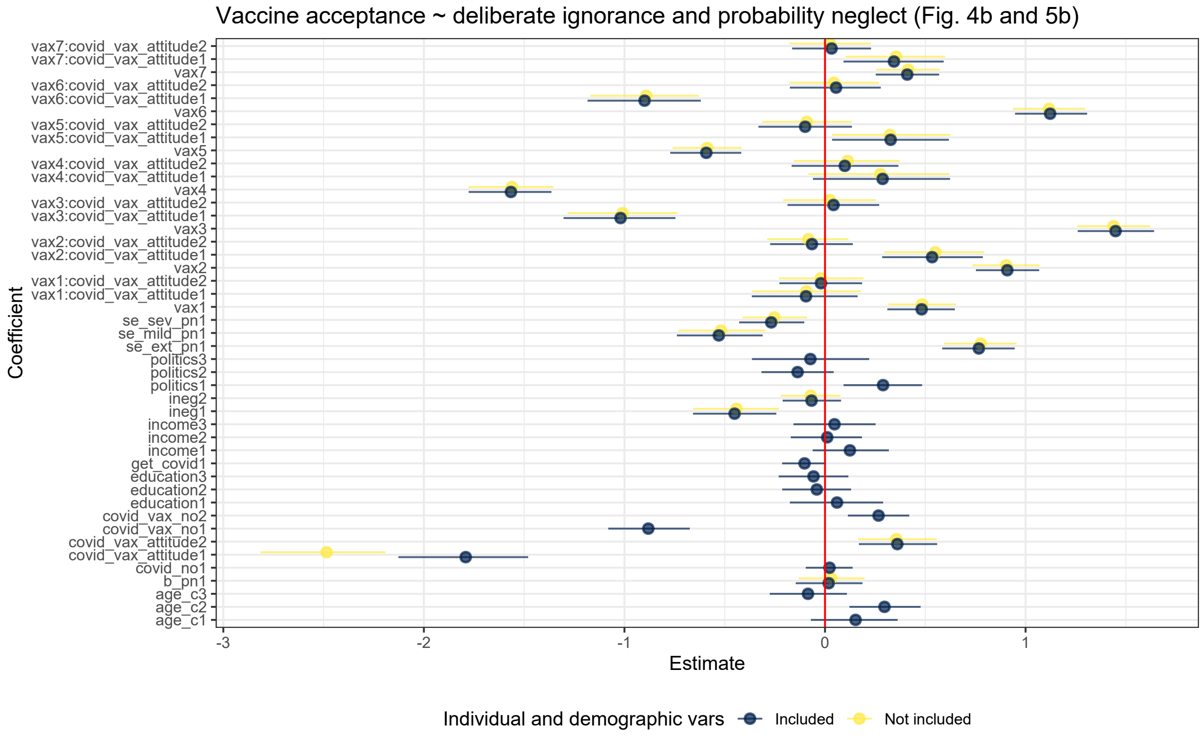

Supplementary Figure 4: Regression weights with 95% HDI for two sets of statistical models, both with vaccination decision as dependent variable. Dark blue shows weights from a model including all listed variables. Yellow shows weights for a model including vaccination attitude, vaccine brand, their interaction, and all indices of deliberate ignorance and probability neglect considered in the main text.

Finally, we ran a specification curve analysis, which involved estimating all possible combinations of regression models built from five predictors (31 models in total): an indicator of deliberate ignorance levels and indicators of four types of attentional probability neglect. These analyses showed that the relationships between vaccination decisions and (1) deliberate ignorance, (2) probability neglect for extreme side effects, and (3) probability neglect

for mild side effects were stable across all possible model specifications (Fig. 5). Thus, the results presented in Figure 4b, and Figures 5b–c in the main text can be considered highly robust.

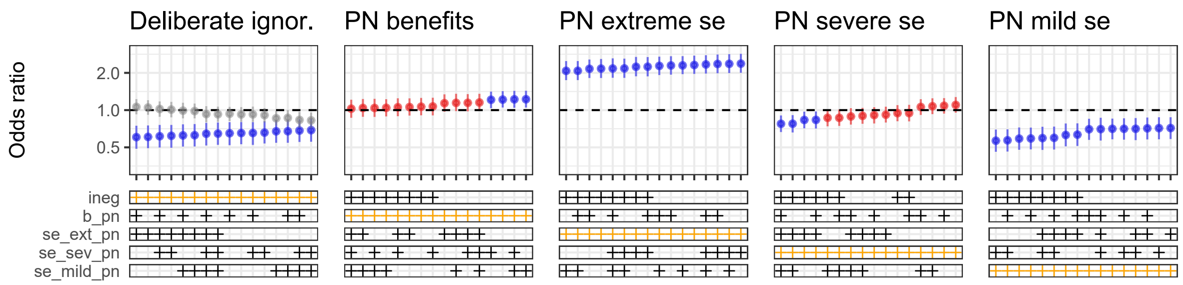

Supplementary Figure 5: Regression weights (in the form of odds ratios) with 95% HDI (points and vertical lines) from Bayesian hierarchical regression model with vaccination decision as dependent variable. Each column of crosses shows which predictor was included in a given regression model. Blue indicates that HDI excludes zero, and red indicates that zero is included. All models included the effects of vaccination attitude, vaccination brand, and the interaction of the two.

Computational Modeling—Alternative Models

Alternative Behavioral Models of Vaccine Decisions

The model presented in the main article was identified as the best-performing model out of a set of alternative models we tested. Specifically, our goal was to build a model able to capture the qualitative patterns in the data and with the best out-of-sample predictive performance in terms of elpd_{loo}. In this section, we provide the full results of the performance of the alternative models, as well as the model reported in the main article.

We started by fitting a model that would typically be used in the context of monetary lotteries—a model consisting solely of the V_{iv} component as given by prospect theory [3] (see Equations 3–5 in the main text), with four individual-level parameters (i.e., with parameters α_i and φ_i also estimated for each participant). In this standard implementation, the probability of accepting a vaccine $P(\text{accept})$ is determined solely by the individual valuation of the vaccine:

$$P(\text{accept}) = \frac{1}{1+\exp -\varphi_i V_{i,v}}. \tag{1}$$

However, this model could not capture the individual- and vaccine-level average proportions of decisions to accept the vaccine, and for the majority of participants, the proportion of correct decision predictions was no higher than the chance level (see Fig. 6). Next, we decided to extend the model to include (i) individual decision bias β_i and (ii) vaccine effects $\sum_j X_v \beta_j$, i.e. vaccine-specific information on country of origin and technology in an attempt to explain the variance that prospect theory itself could not account for. We also decided to estimate α and φ only on the population level to reduce the number of individual-level parameters since these parameters were not of theoretical interest. The resulting model captured the qualitative patterns at the individual- and vaccine level well and could predict individual decisions above chance level for nearly all participants (see Fig 7). Thus, this is the model we presented and used for inference in the main paper. The model also outperformed other plausible versions (Fig. 8), which we will discuss in more detail next.

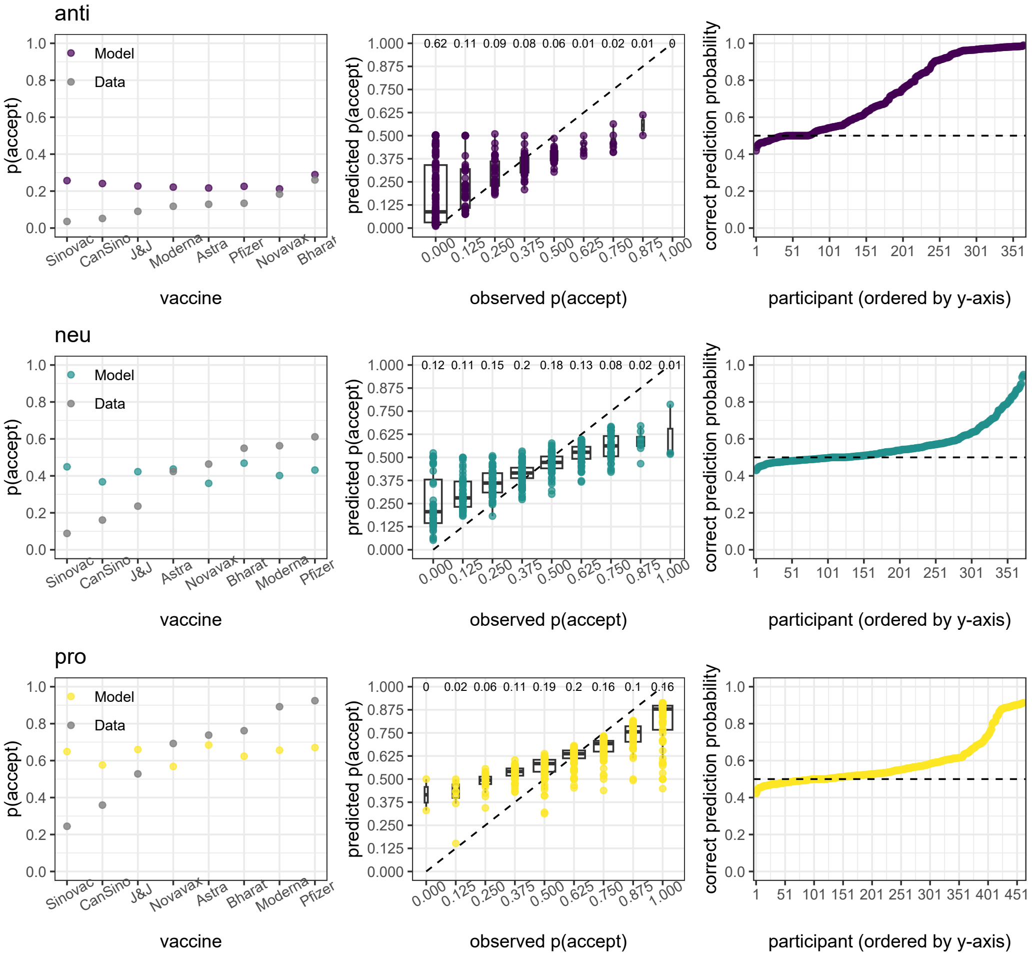

Supplementary Figure 6: Qualitative and predictive performance evaluation of a standard implementation of prospect theory, separately for each vaccine attitude group (rows). The first column shows observed and predicted (i.e., derived from the model) proportions of decisions to accept each vaccine. The second column shows the relationship between observed and predicted individual-level proportions of accepted decisions. The values on the top of each panel show the proportion of participants with a given proportion of accepted decisions. Note that the proportions on the x-axis correspond to accepting between zero to all eight vaccines presented to each participant. The third column shows individual-level proportions of vaccination decisions correctly predicted by the model (based on approximate out-of-sample predictions). The dashed line shows chance level prediction.

A series of model comparisons were made to test whether two simpler but plausible models could account for the data we conducted. The first could be called a *decision bias* model, as it only included the first two components from Equation 1 in the main text. It thus assumes that participants ignored all information on vaccine outcomes and their probabilities. The second model, which we refer to as the *outcome heuristic*, assumes that participants used outcome information to inform their vaccination decisions but ignored all probability information. Formally, in the outcome heuristic the $V_{i,v}$ component was:

$$V_{i,v} = \sum_{se} v(a^i_{se}) + \sum_{b} v(a^i_{b}), \tag{2}$$

where the value function was as in Equation 4 in the main text. Thus, the outcome heuristic was equivalent to setting all decision weights in Equation 3 to $w(p) = 1$, which would be an alternative instance of probability neglect—i.e., ignoring all probability information even after inspecting it.

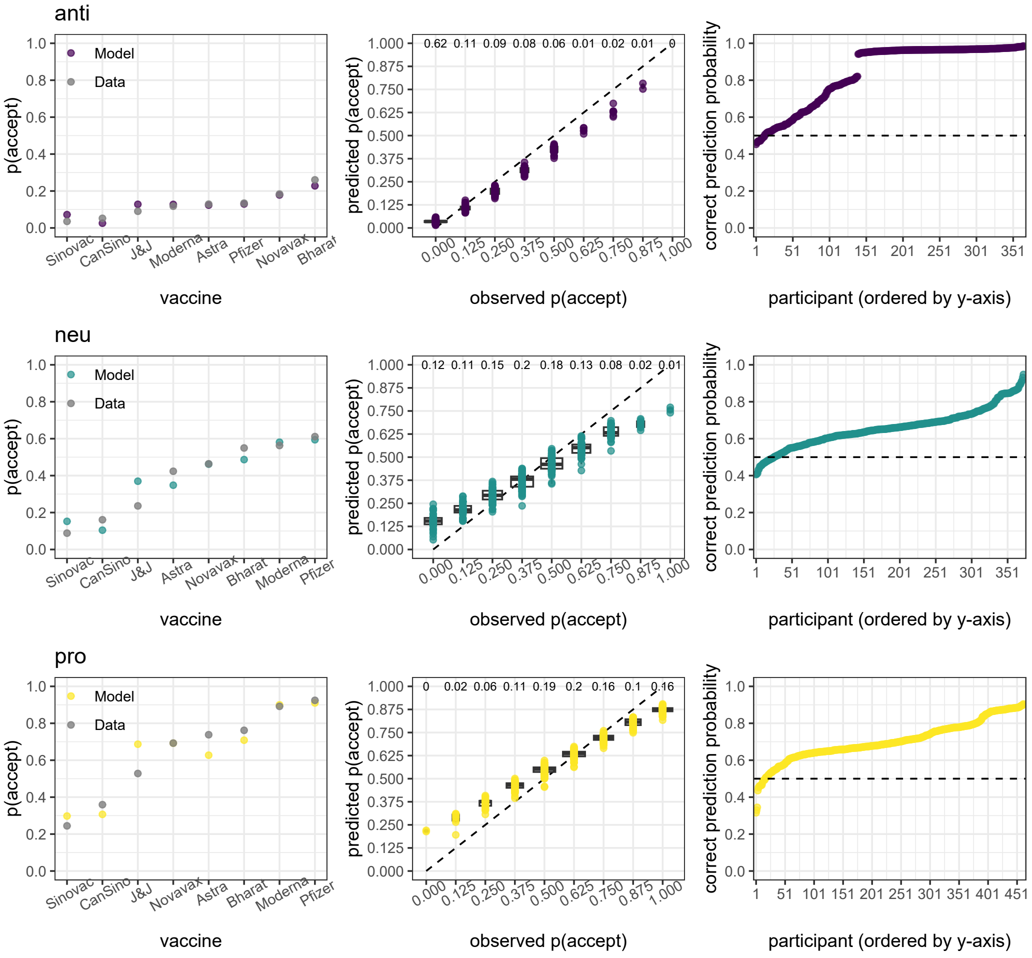

Supplementary Figure 7: Qualitative and predictive performance evaluation of an implementation of prospect theory extended with vaccine-effects and individual bias (i.e., the model presented in the main text), separately for each vaccine attitude group (rows). The first column shows observed and predicted (i.e., derived from the model) proportions of decisions to accept each vaccine. The second column shows the relationship between observed and predicted individual-level proportions of accepted decisions. The values on the top of each panel show the proportion of participants with a given proportion of accepted decisions. Note that the proportions on the x-axis correspond to accepting between zero to all eight vaccines presented to each participant. The third column shows individual-level proportions of vaccination decisions correctly predicted by the model (based on approximate out-of-sample predictions). The dashed line shows chance level prediction.

Comparing the out-of-sample predictive performance of these three models provides additional valuable insights into the information processing of the three groups (Fig. 8). First, the decision bias model was the worst-performing model in all three groups, indicating that all participants used outcome and probability information to inform their vaccination decisions to at least some degree. Second, the performance increase from the decision bias model through the outcome heuristic to extended prospect theory was most significant in the neutral group, indicating that participants in this group were the most sensitive to the outcome and probability information. Third, the performance increase from the outcome heuristic to prospect theory in the anti-vaccination group was significant but marginal, suggesting that these participants exhibited alternative probability neglect (i.e., ignored probability information even after inspecting it). Finally, in the anti-vaccination group, the $V_{i,v}$ component of both the outcome heuristic and prospect theory improved performance relative to the response bias model only for the small proportion of the data associated with vaccine acceptance decisions. These results have two implications: (1) vaccine

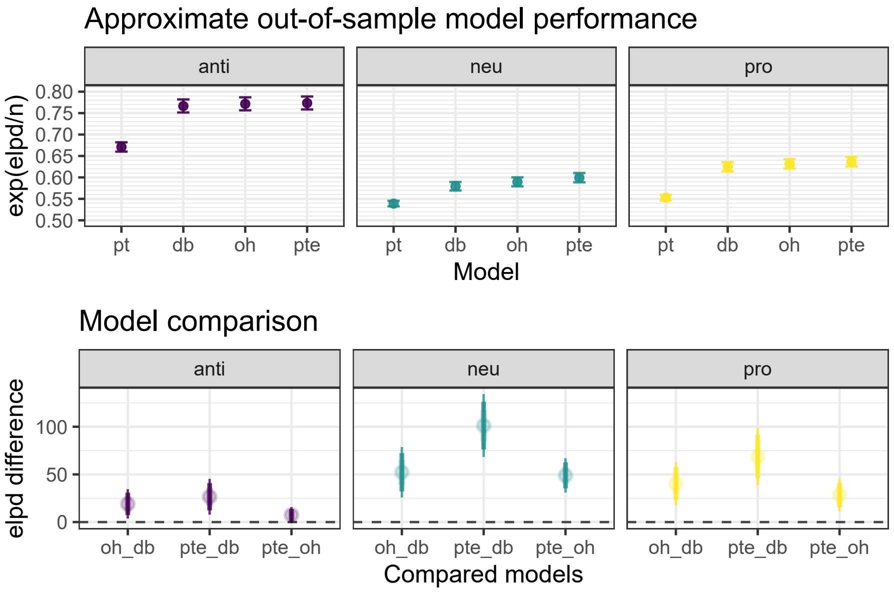

Supplementary Figure 8: Top row shows approximate of-of-sample model performance, separately for attitude groups. The statistic on the y-axis is a nonlinear transformation of elpd_{loo} statistic (see main text)—the value of 0.5 means the expected model performance is no better than chance. In contrast, a value of 1 means the model predicts each decision perfectly. The bottom row shows the results of the model performance comparison. The points are differences in elpd_{loo} between pairs of models, with the vertical line showing 99% confidence intervals—the performance of any two models can be considered significantly different when confidence intervals exclude zero. Note: pt - standard implementation of prospect theory; db - decision bias model; oh - outcome heuristic model; pte - prospect theory. The db model also included vaccine effects, and the oh and pte models included individual bias and vaccine effects components. See text for details.

refusal decisions in the anti-vaccination group might be driven by factors other than those included in our model since the majority of them are captured solely by decision bias, and (2) the value and weighting functions estimated in this group represent the minimum level of loss aversion and probability sensitivity required to overcome the strong vaccine refusal biases in this group.

It is important to note that due to high vaccine refusal rates in the anti-vaccination group, the estimation of the individual- and group-level parameters capturing loss aversion and probability sensitivity is associated with much greater levels of uncertainty in that group than in the neutral and pro-vaccination groups. Nevertheless, parameter recovery analyses show that the population-level parameters λ, γ, and β, on which we based our inference (i.e., loss aversion, probability sensitivity, and decision bias), can be recovered satisfactorily (Fig. 9).

Alternative Implementations of Probability Weighting Function

The second case of Equation 5 in the main text refers to probability neglect—a situation in which an outcome was inspected but not its corresponding probability. For such cases, we set $w(p) = .5$, which means that the decision-maker acknowledges the probabilistic nature of the inspected outcome. Two alternative and strong assumptions could be made. Instead, the first is $w(p) = 0$, which would mean that the decision-maker perceives the outcome of the neglected probability as impossible; the second strong case would be $w(p) = 1$, meaning that the decision-maker perceives the outcome as certain to occur. Assuming any value in the 0–1 range is also possible. For this reason, we also tested model versions that treated the value of the neglected probabilities as free parameters.

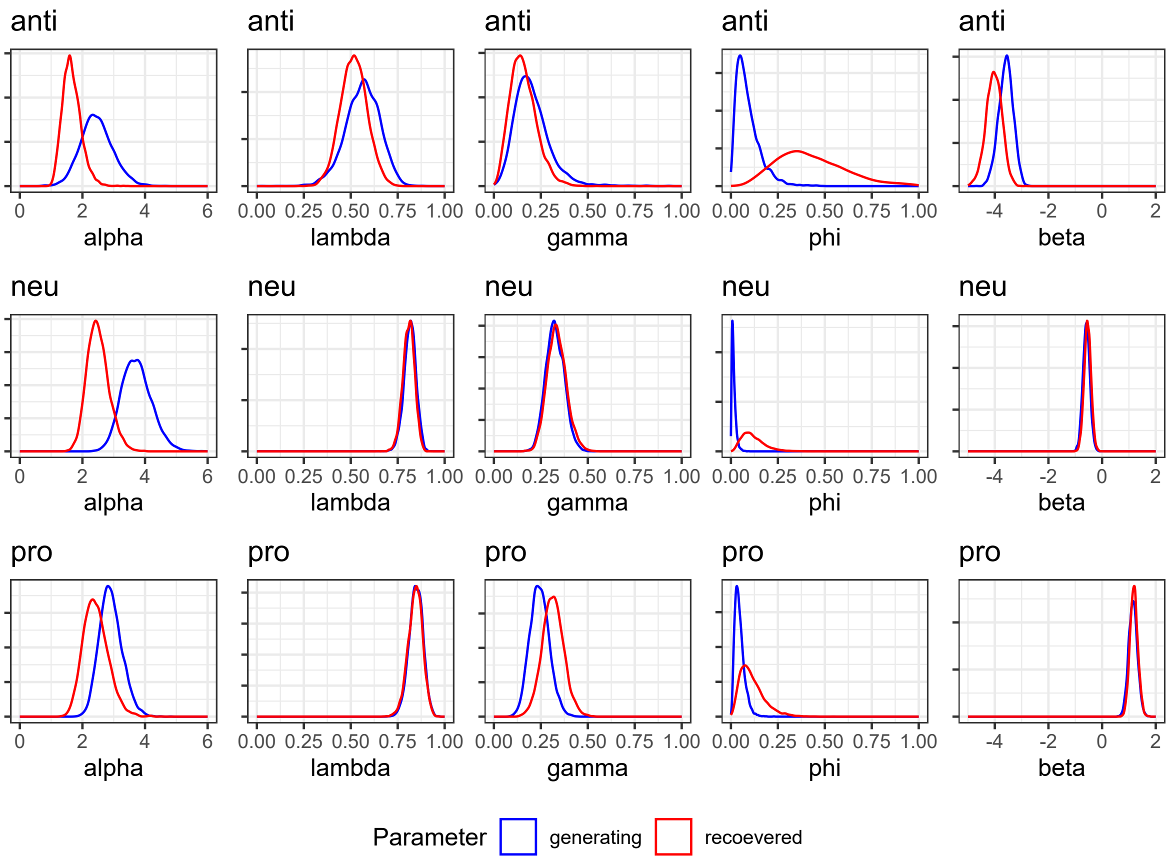

Supplementary Figure 9: Main results of the parameter recovery analyses result, conducted separately for each attitude group (rows). The first step of the analysis was to simulate individual decisions using mean posterior values of the model parameters, including individual-level parameters—these constitute the *generating* parameters. Next, the model parameters are recovered—i.e., the model is fitted to the simulated set of decisions to obtain *recovered* parameter estimates. Finally, the posterior distribution of the population-level generating and recovered parameters are compared—the results can be considered satisfactory if the recovered posterior estimates are qualitatively similar to the generating posteriors.

However, the main conclusions from these models were qualitatively the same as those reported in the main text, and model performance, relative to the model assuming $w(p) = 0.5$, improved only slightly in the neutral group.

Another important assumption that we make is that people treated vaccine effectiveness as the probability of being protected from COVID-19 infection, severe illness, or death, but technically the numbers provided are not probabilities. The vaccine effectiveness reported in the study was defined and explained to participants as $(1 - RR) \times 100\%$, where RR is the relative risk of developing an infection in the vaccinated group relative to the unvaccinated group. Vaccine effectiveness was always in the 0.5–1 range for the vaccines presented in our study, so it constituted a valid input to the probability weighting function. Importantly, people often misperceive the effectiveness of vaccination in terms of the probability of not getting infected after a vaccination [4], which, at least to some extent, justifies our assumption.

Supplementary References

1. Gabry, J., Simpson, D., Vehtari, A., Betancourt, M. & Gelman, A. Visualization in Bayesian workflow. *Journal of the Royal Statistical Society Series A: Statistics in Society* **182,** 389–402 (2019).

2. Vehtari, A., Gelman, A. & Gabry, J. Practical Bayesian model evaluation using leave-one-out cross-validation and WAIC. *Statistics and Computing* **27,** 1413–1432 (2017).

3. Tversky, A. & Kahneman, D. Advances in prospect theory: Cumulative representation of uncertainty. *Journal of Risk and Uncertainty* **5,** 297–323 (1992).

4. Tentori, K., Passerini, A., Timberlake, B. & Pighin, S. The misunderstanding of vaccine efficacy. *Social Science & Medicine* **289,** Article 114273 (2021).
